# Efficient Synthesis of 2’‐*O*‐Methoxyethyl Oligonucleotide‐Cationic Peptide Conjugates

**DOI:** 10.1002/cmdc.202100388

**Published:** 2021-09-08

**Authors:** François Halloy, Alyssa C. Hill, Jonathan Hall

**Affiliations:** ^1^ Department of Chemistry and Applied Biosciences ETH Zurich Vladimir Prelog Weg 4 8093 Zurich Switzerland; ^2^ Current address: Department of Paediatrics Medical Sciences Division University of Oxford OX1 3QX Oxford UK

**Keywords:** oligonucleotides, cell-penetrating peptides, bioconjugation, albumin conjugation, mass spectrometry

## Abstract

Single‐stranded phosphorothioate (PS) oligonucleotide drugs have shown potential for the treatment of several rare diseases. However, a barrier to their widespread use is that they exhibit activity in only a narrow range of tissues. One way to circumvent this constraint is to conjugate them to cationic cell‐penetrating peptides (CPPs). Although there are several examples of morpholino and peptide nucleic acids conjugated with CPPs, there are noticeably few examples of PS oligonucleotide‐CPP conjugates. This is surprising given that PS oligonucleotides presently represent the largest class of approved RNA‐based drugs, including Nusinersen, that bears the 2’‐*O*‐methoxyethyl (MOE)‐chemistry. In this work, we report a method for in‐solution conjugation of cationic, hydrophobic peptides or human serum albumin to a 22‐nucleotide MOE‐PS oligonucleotide. Conjugates were obtained in high yields and purities. Our findings pave the way for their large‐scale synthesis and testing *in vivo*.

## Graphical abstract

**Broadening horizons**: Oligonucleotide therapeutics are an emerging class of drugs. Their delivery into cells can be enhanced by covalent conjugation to short, cationic cell‐penetrating peptides. The synthesis of such conjugates is challenging because of molecular aggregation. Herein we report an efficient methodology to access oligonucleotide conjugates of the clinically validated 2’‐*O*‐methoxyethyl modification, including key conjugation parameters.

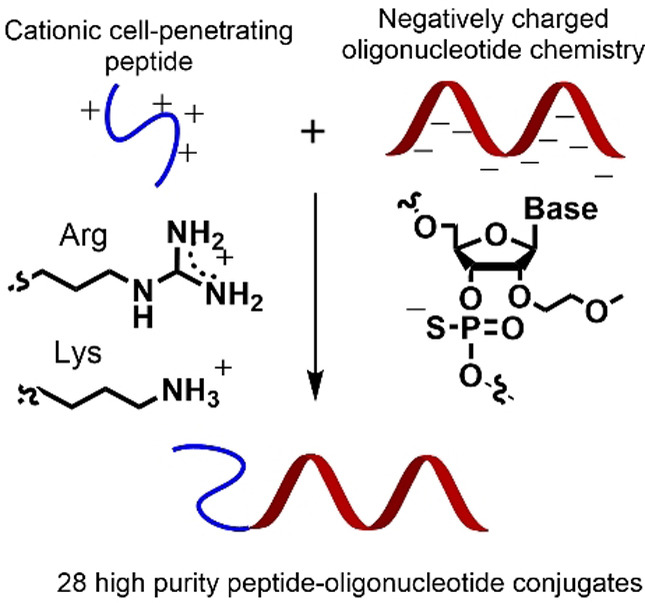

The 2’‐*O*‐methoxyethyl (2’‐MOE) ribose chemistry (Figure 1A)^[1]^ is a widely used oligonucleotide modification which, in combination with a PS backbone, has been successfully employed in many clinically approved oligonucleotide drugs, including Nusinersen^[2]^ and Inotersen.^[3]^ Compared to other oligonucleotide modifications, 2’‐MOE PS oligonucleotides display enhanced binding affinity to their target RNAs, useful binding to serum proteins and an excellent resistance to ubiquitous nucleases *in vivo*.^[4]^ However, a common drawback for this class of molecules is that they demonstrate useful pharmacological activity in only a few tissues.^[5]^

**Figure 1:**
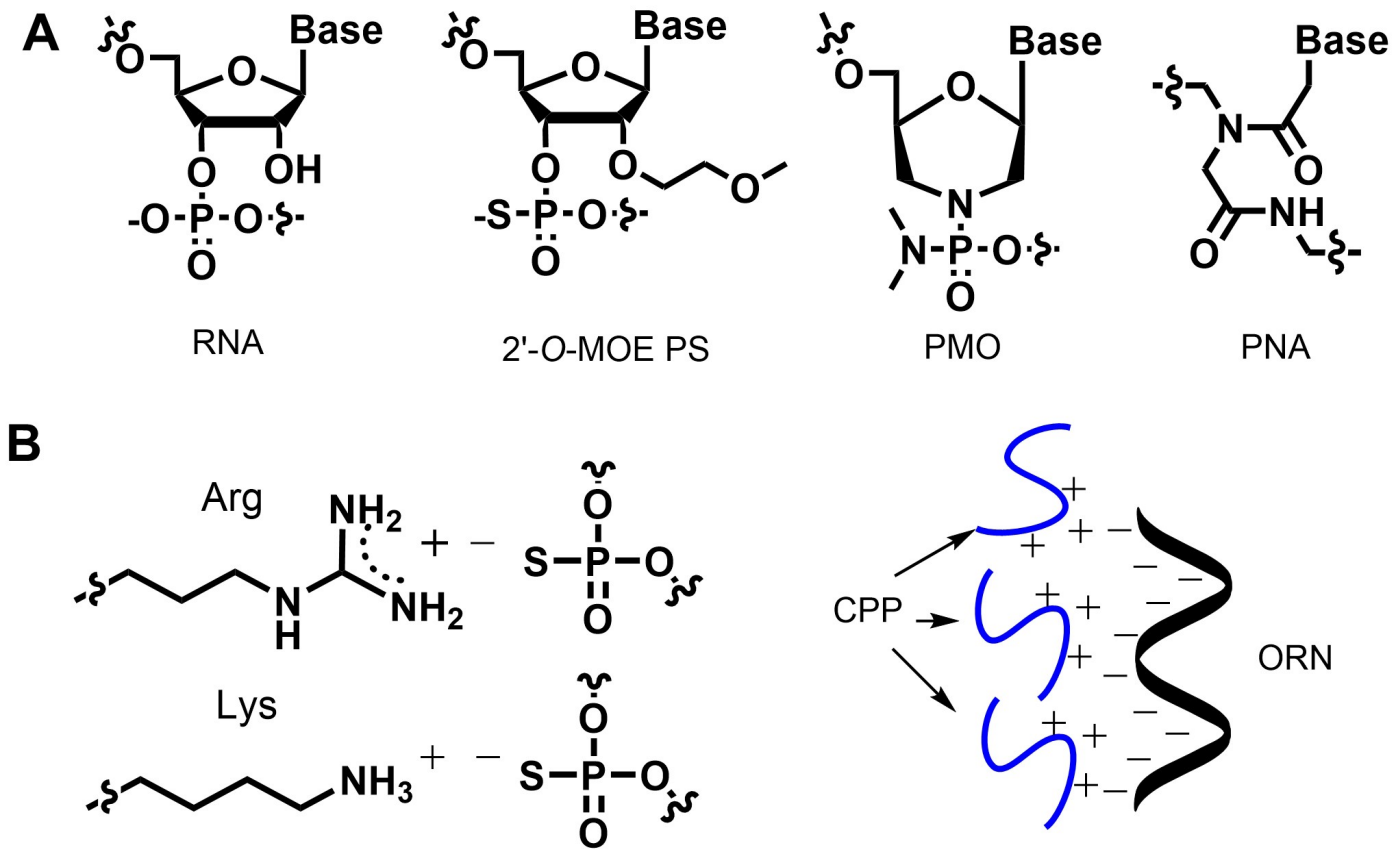
*Oligonucleotide chemistries used in the synthesis of peptide‐oligonucleotide conjugates*. **A** RNA‐based drugs require chemical modifications for metabolic stability. The 2’‐*O*‐MOE PS modification is negatively charged due to a phosphorothioate (PS) backbone unit. Uncharged chemistries include the PMO and PNA chemistries. **B** PS‐ and PO‐oligonucleotides are prone to aggregation with cationic CPPs owing to electrostatic interactions between arginine and lysine residues and the oligonucleotide backbone. CPP: cell‐penetrating peptide; ORN: oligoribonucleotide.

One means to enhance tissue‐selective oligonucleotide delivery and cellular uptake is *via* conjugation to short peptide sequences that cross cellular membranes or bind to cell membrane receptors and trigger internalization. Such cell‐penetrating peptides (CPPs) are typically 5–30 amino acids long and are generally cationic at physiological pH owing to the presence of arginine and lysine residues important for cellular uptake.^[6]^ Two promising receptor binding peptides are the weakly charged glucacon‐like 1 receptor peptide for delivery to pancreatic cells^[7]^ and the neurotensin peptides for use in the central nervous system.^[8]^ Most of the current research on cationic CPPs is with non‐PS oligonucleotide chemistries: the uncharged peptide nucleic acids (PNAs),^[9]^ for which peptides are conjugated directly during solid‐phase synthesis, and phosphorodiamidate morpholino (PMO) oligonucleotides,^[10]^ which have an uncharged backbone (Figure 1A). With a few exceptions,^[11]^ oligonucleotides with phosphodiester (PO) or PS backbones are rarely conjugated with CPPs, because: (i) the CPP fragment is not fully resistant to the harsh basic conditions of oligonucleotide deprotection,^[12]^ and (ii) in solution, the positively charged CPPs associate with the negatively charged oligonucleotide backbone causing aggregation and difficulties with product isolation and purification (Figure 1B; references in^[13]^).

We selected 14 peptide moieties for conjugation to PS‐oligonucleotides with the aim to improve cellular uptake,[^9^, ^11a^, ^14^] endosomal escape,^[15]^ cell membrane receptor binding,^[16]^ nuclear localization^[17]^ or delivery into the bone marrow compartment *in vivo*.^[18]^ We examined oligonucleotide conjugation to either the N‐terminus or the C‐terminus for each peptide, since peptide orientation may impact the pharmacokinetic properties of the conjugate^[11a]^ (Table 1). As a test sequence, we selected a 22‐mer 2’‐MOE‐PS oligonucleotide that we have used previously to modulate splicing of the *FECH* pre‐mRNA in the context of erythropoietic protoporphyria (EPP),^[19]^ a rare disease caused by accumulation of the toxic heme precursor protoporphyrin IX in red blood cells.^[20]^

**Table 1:** *List of 2’‐O‐MOE conjugates produced by thiol‐maleimide addition*.

Conjugate ID^[a]^	Peptide sequence N→C^[a]^	Charge (pH 7)	MW peptide/ conjugate [%]	Peptide hydrophobic moment^[b]^	ΔG [kcal.mol^−1^]^[c]^	Conjugation buffer^[d]^	Yield [%]^[e]^	Purity [%]^[f]^
**1**	CRKKRRQRRRPPQ	+9	16.7	2.67	−22.56	A	55	>99
**2**	RKKRRQRRRPPQC	+7	17.1	2.65	−23.86	A	68	>99
**3**	CRQIKIWFQNRRMKWKKGG	+8	21.9	6.95	−16.8	A	45	94
**4**	RQIKIWFQNRRMKWKKGGC	+6	22.2	6.93	−18.1	A	38	>99
**5**	CRRRRRRRQIKIWFQNRRMKWKKGG	+14	27.9	4.9	−27.66	C	41	>99
**6**	RRRRRRRQIKIWFQNRRMKWKKGGC	+13	27.9	4.9	−31.26	C	19	>99
**7**	CGLFHAIAHFIHGGWHGLIHGWYG	+0.5	23.4	19.13	−9.66	A+μλ	3	54
**8**	GLFHAIAHFIHGGWHGLIHGWYGC	+0.5	23.5	19.14	−13.26	A+μλ	n.d.	n.d
**9**	CHAIYPRH	+1	10.2	0.9	−13.16	A	37	>99
**10**	HAIYPRHC	+1	10.2	0.87	−13.16	A	43	>99
**11**	CTHRPPMWSPVWP	+1	15.4	5.16	−7.98	A	65	89
**12**	THRPPMWSPVWPC	+1	15.4	5.13	−7.98	A	50	96
**13**	CRRRRRRRRRFF	+9	17.2	4.56	−20.75	A	40	96
**14**	RRRRRRRRRFFC	+9	17.2	4.53	−20.75	A	39	>99
**15**	CVQRKRQKLMP	+5	13.6	4.38	−12.8	A	48	76
**16**	VQRKRQKLMPC	+3	14.0	4.39	−14.1	A	57	75
**17**	CSKKKKTKV	+6	10.7	1.45	−18.53	A	71	>99
**18**	SKKKKTKVC	+4	11.1	1.48	−19.83	A	68	>99
**19**	CPKKKRKV	+6	10.1	3.47	−16.97	A	26	90
**20**	PKKKRKVC	+4	10.5	3.47	−18.27	A	33	89
**21**	CQEQLERALNSS	−1	13.6	8.42	−18.26	A or D	62	94
**22**	QEQLERALNSSC	+1	13.6	8.4	−18.26	A or D	45	86
**23**	CESGGGGSPGRRRRRRRRRRR	+10	22.4	3.21	−38.23	A	10	>99
**24**	ESGGGGSPGRRRRRRRRRRRC	+10	22.4	3.22	−38.23	A	32	>99
**25**	CNDTIPEDFQEFQTQNFDRFDN	−5	23.7	19.07	−29.05	A	19	91
**26**	NDTIPEDFQEFQTQNFDRFDNC	−6	24.0	19.09	−26.75	A	26	>99
**27**	CSTFTKSP	+1	9.0	4.67	−10.53	A or D	63	>99
**28**	STFTKSPC	+1	9.0	4.65	−10.53	A or D	38	>99

[a] Cys for conjugation is either at N or C terminus. [b], [c] calculated from MPex.^[24]^ [c] is the peptide transfer free energy from water to octanol. [d] Buffer A: 0.3 M KBr, 8 M urea, 0.1 M KH_2_PO_4_ pH 6.8; Buffer C: 50 % DMA, 30 mM KBr, 0.8 M urea, 10 mM K_2_HPO_4_ pH 6.8; Buffer D: 20 % DMA, 0.33 M TEAA pH 8; μλ: microwave irradiation for 30 min at 1 W. [e] Yield was calculated by Nanodrop measurement of the final material after conjugation, purification and ultracentrifugation. [f] Area‐under‐the‐curve of product peak in final LC‐MS chromatogram. Spectra are provided in Supporting Information. MW=Molecular Weight.

We employed a solution phase thiol‐maleimide conjugation protocol for the preparation of structurally diverse oligonucleotide conjugates. Thiol‐maleimide chemistry is used in several antibody‐drug conjugates that have been approved by regulatory authorities.^[21]^ A cysteine amino acid was added to the terminal position of each peptide to provide the S‐nucleophile, which reacts readily with the maleimide group. The 5’‐masked maleimide PS‐MOE oligonucleotide was prepared in solid‐phase under standard conditions using a commercially available building block (Scheme 1). Since the masked maleimide group is unstable in ammonia at high temperatures (Figure S1), oligonucleotide deprotection and cleavage from the solid support was carried out at 35 °C. The maleimide modifier was then unmasked by microwave irradiation in water (see references in^[22]^). Next, we aimed to identify optimal conjugation‐ and purification‐protocols for the preparation of a library of oligonucleotide conjugates.

**Scheme 1:**
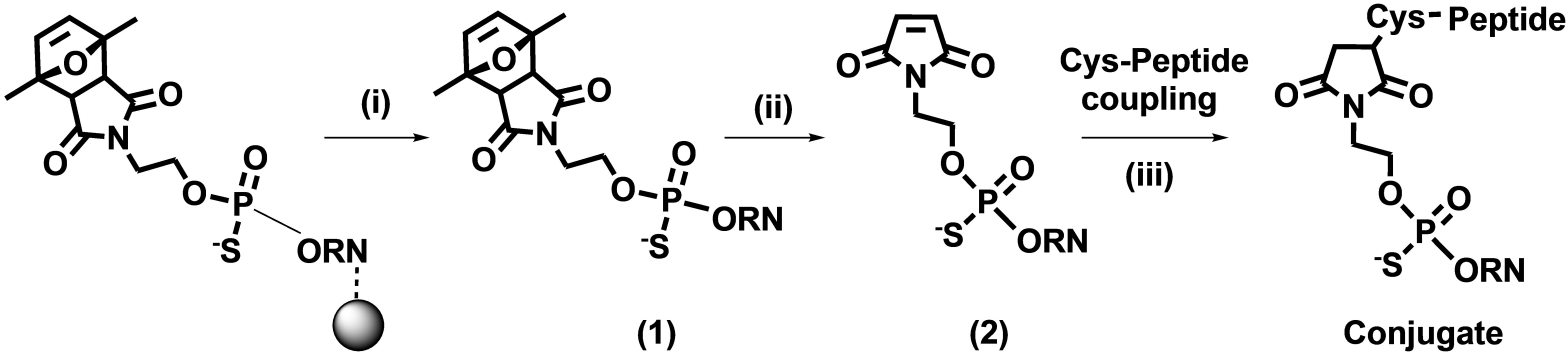
*Synthetic route to 2’‐O‐MOE PS conjugates*. Conjugates were produced in a three‐step protocol. The 5’‐maleimide “modifier” was introduced during oligonucleotide solid‐phase synthesis. Nucleobase deprotection and cleavage from solid support yielded Intermediate (**1**) with a 5’‐protected maleimide. The maleimide was then uncaged by microwave irradiation to give (**2**) and coupled in solution to a peptide containing a terminal cysteine. (i) 25 % ammonia, 35 °C, overnight. (ii) H_2_O, 90 °C, 90 min, microwave irradiation (iii) 6 molar equivalents Cys‐peptide, buffers A, B, C or D for 1 h. See also Supporting Information. CPG: controlled pore glass solid support.

In order to avoid aggregation of oppositely‐charged oligonucleotides and peptides during the various synthesis and purification steps, we employed buffers containing denaturing agents such as urea^[14a]^ or formamide.^[11a]^ We compared two denaturing solvent systems for the conjugation of the moderately charged SV40 peptide (presumably +6‐charged at pH 7) and subsequent purification (Entry **19** in Table 1; procedures and reagents are described in Supporting Information). In the first, we examined conjugation in a buffer of K_2_HPO_4_/KBr/urea (Buffer A), followed by IE‐HPLC using the same system but with a concentration gradient of aqueous (aq.) KBr. In the second, we carried out conjugation in 70 % formamide/triethylammonium acetate (TEAA) (Buffer B), followed by IE‐HPLC using the same system but with a concentration gradient of aq. NaClO_4_ or NaCl. Both protocols yielded the desired product **19** in high purity (Figure S2). Since the isolated yield was highest with the urea‐containing system, we subsequently purified all cationic CPPs of charge ≥2 using this protocol. Products that we considered of a low likelihood for aggregation (charge −1 to +1, at pH 7), were purified by reverse‐phase (RP) HPLC (Table 1).

In all, 27 oligonucleotide‐peptide conjugates were recovered in yields up to 71 %, with UV‐purities routinely in excess of 90 % (Table 1). Of note, we succeeded in producing the highly cationic Tat peptide conjugates **1** and **2** with yields of 58 and 65 %, respectively, and the R6‐Penetratin peptide conjugates **5** and **6** in yields of 19 and 41 %; the four products exhibited excellent purities (Figure 2; Supporting Information). The conjugates **5** and **6** were not obtained using Buffer A, but were produced using a more apolar solvent system (50 % dimethylacetamide (DMA), 30 mM KBr, 0.8 M urea, 10 mM K_2_HPO_4_ pH 6.8: Buffer C). The hydrophobic H5WYG peptide conjugates **7** and **8** proved the most difficult to prepare. These were not obtained using Buffers A or C, nor in aqueous DMF or acetonitrile. The conjugate **7** was finally isolated in low yield under microwave irradiation in Buffer A. The lowest product purities were obtained using methionine‐containing peptides **15** and **16**, where partial methionine oxidation was observed. After HPLC purification, the conjugates were ultracentrifuged three times in ultrapure water prior to calculation of purity and yield (Table 1; Figures S6–S30).

**Figure 2:**
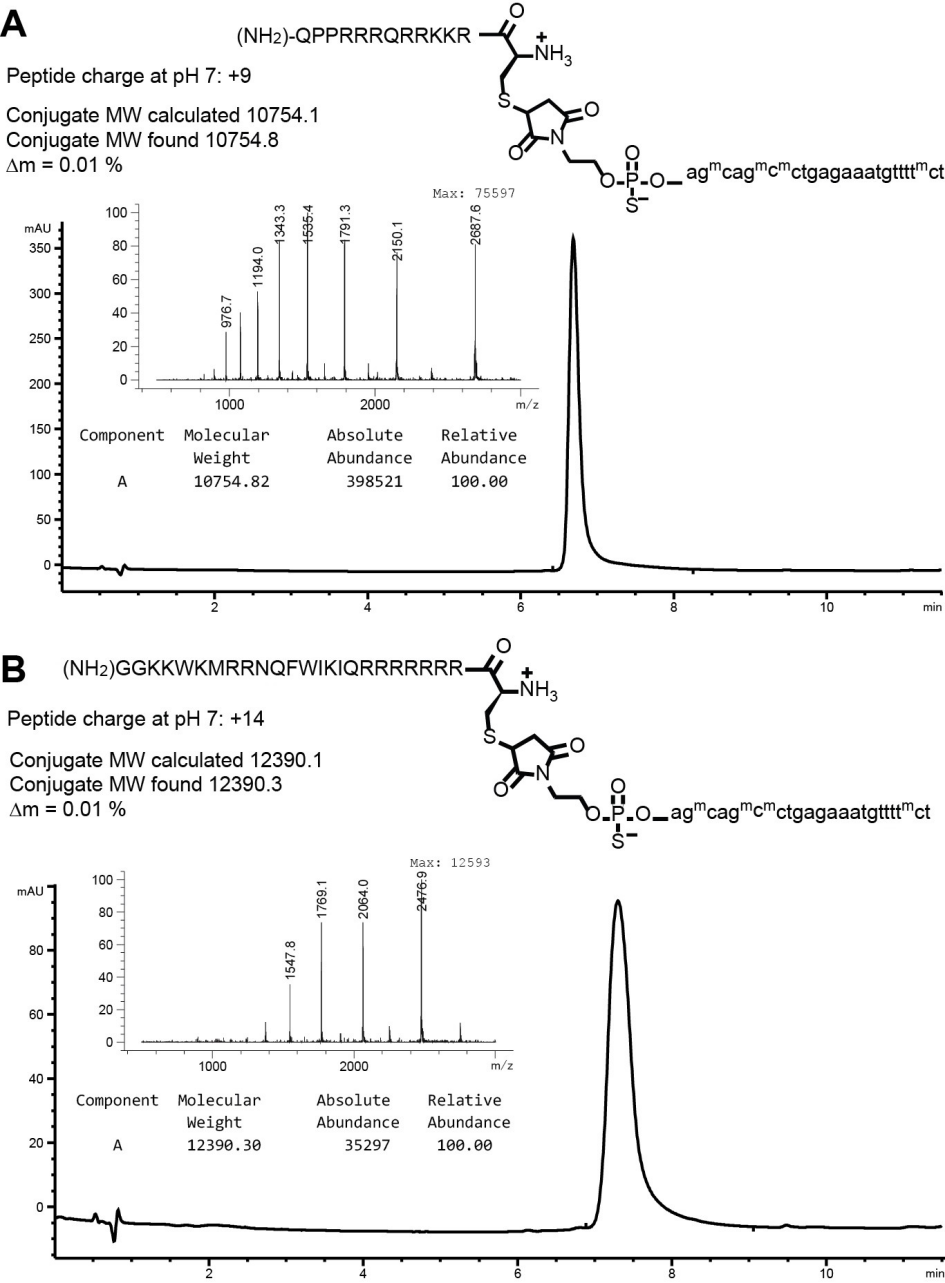
Chromatograms *of purified cationic 2’‐O‐MOE PS conjugates*. Cationic conjugates **1** (**A**) and **5** (**B**) were analysed by LC‐MS (Agilent 1200/6130 system) on a Waters Acquity OST C18 column, at 65 °C with a gradient of methanol in 0.4 M HFIP, 15 mM trimethylamine. ^m^C: 5‐methyl 2’‐*O*‐methoxyethyl cytosine.

Next, we searched for possible correlations between conjugation yields and peptide properties. We first determined the relative lipophilicity of the conjugates after injection into a C18 reverse phase column (Figure S3). The retention times of the macromolecules was used as a measure of their lipophilicity, as previously reported.^[23]^ Surprisingly, we observed only minor differences in the retention times of the conjugates and the parent 5’ maleimide oligonucleotide, but far from those observed after conjugation of the oligonucleotide to hydrophobic moieties such as stearic acid or cholesterol (Figure S3[^19^, ^23^]).

Then we used MPex, a tool developed by Snider, White and coworkers^[24]^ for the study of membrane proteins. MPex predicts properties of unfolded peptide sequences based on their sequence, e. g. the free energy (ΔG) of transfer from water to a hydrophobic phase (octanol), as well as the hydrophobic moment. We compared the synthesis yields of the conjugates with: (i) the peptide net charge at pH 7, (ii) the peptide molecular weight, (iii) the peptide hydrophobic moment and, (iv) the ΔG of peptide transfer from water to octanol. For each measurement, we calculated a Pearson correlation coefficient (Figure 3). We observed no correlation between the synthesis yield and the charge or hydrophobicity of the peptides (Figure 3A–B). However, we did observe a correlation between the yield and the peptide molecular weight (Figure 3C), as well as between the yield and the ΔG of peptide transfer from water to octanol (Figure 3D). Although the analysis was limited by the number and diversity of peptide sequences, the results suggested that it may be possible to predict conjugation efficiency based on peptide molecular weight or free energy of transfer calculations, an observation which has not previously been described.

**Figure 3:**
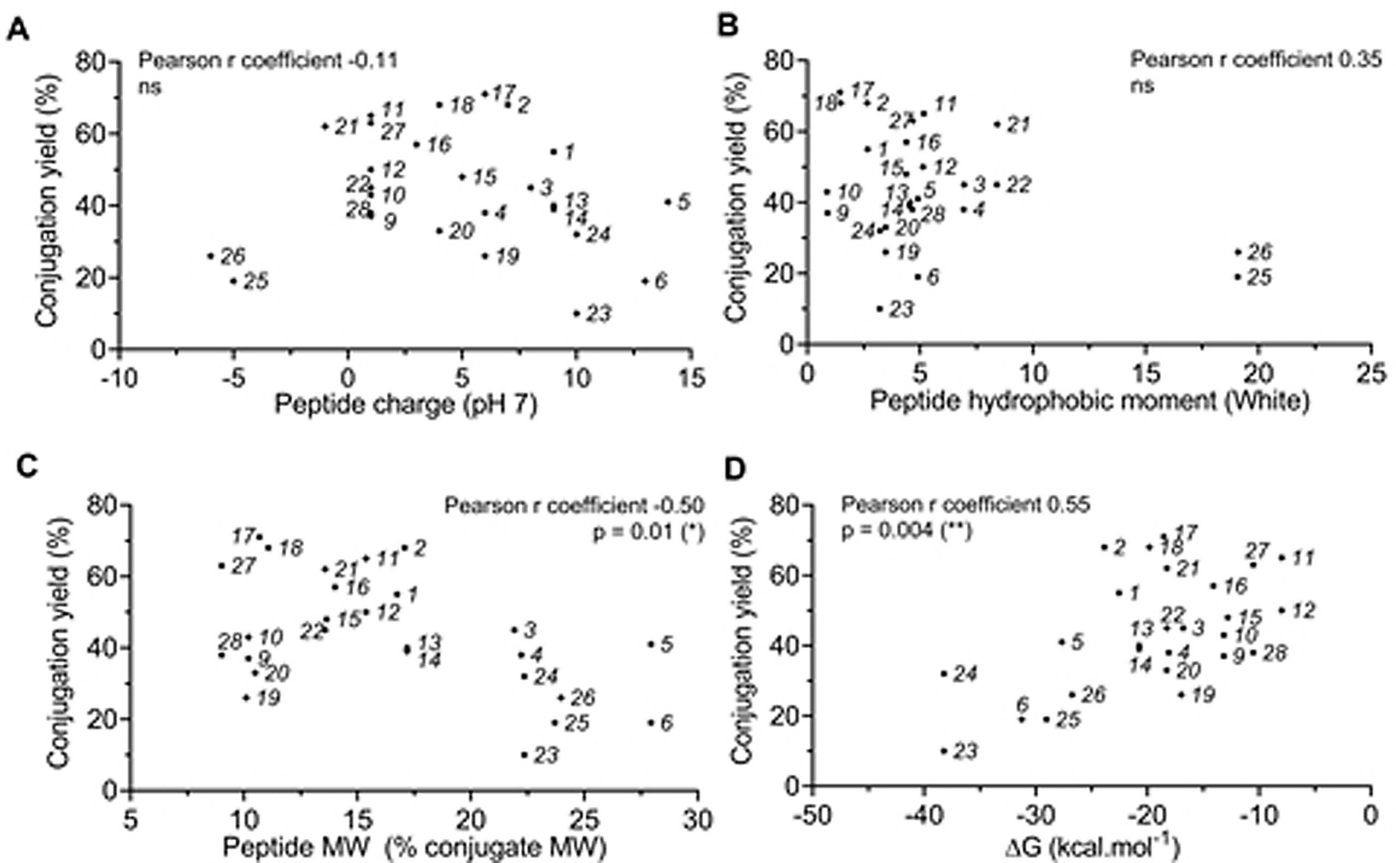
*Analysis of conjugation yield versus selected peptide characteristics*. Yields of oligonucleotide‐peptide conjugates from Table 1 were plotted against peptide charge (**A**), hydrophobic moment (**B**), molecular weight (**C**) or free energy (ΔG) of transfer from water to octanol (**D**). Hydrophobic moment and ΔG values were calculated from MPex,^[24]^ peptide charge from PepCalc.^[29]^ Pearson correlation tests were performed for each comparison, and the p value is the probability that the correlation is due to random sampling. ns: not significant.

Most peptides exhibited calculated hydrophobicities of between 2–8, according to the scale of White et al^[24]^ (Table 1). This included the CPPs Tat and Penetratin, which have been used in many uptake studies.[^11a^, ^25^] The anionic CPPs of conjugates **25** and **26** showed high calculated hydrophobicities of approximately 20.

In a final stage, we tested the synthesis protocol with the synthesis of a 2’‐*O*‐MOE PS protein conjugate. As a model, we employed human serum albumin (HSA; 66 kDa), which bears a single cysteine at amino acid position 34.^[26]^ Conjugation of HSA to oligonucleotides is of potential interest since the protein has a long half‐life in the bloodstream^[27]^ and retards the nuclease degradation of oligonucleotides to which it binds non‐covalently.^[28]^ We performed the conjugation in 20 % dimethylacetamide, 0.33 M TEAA pH 8 (Buffer D). The HSA conjugate was resolved from unconjugated albumin by RP‐HPLC, and was obtained in high purity (Figure 4).

**Figure 4:**
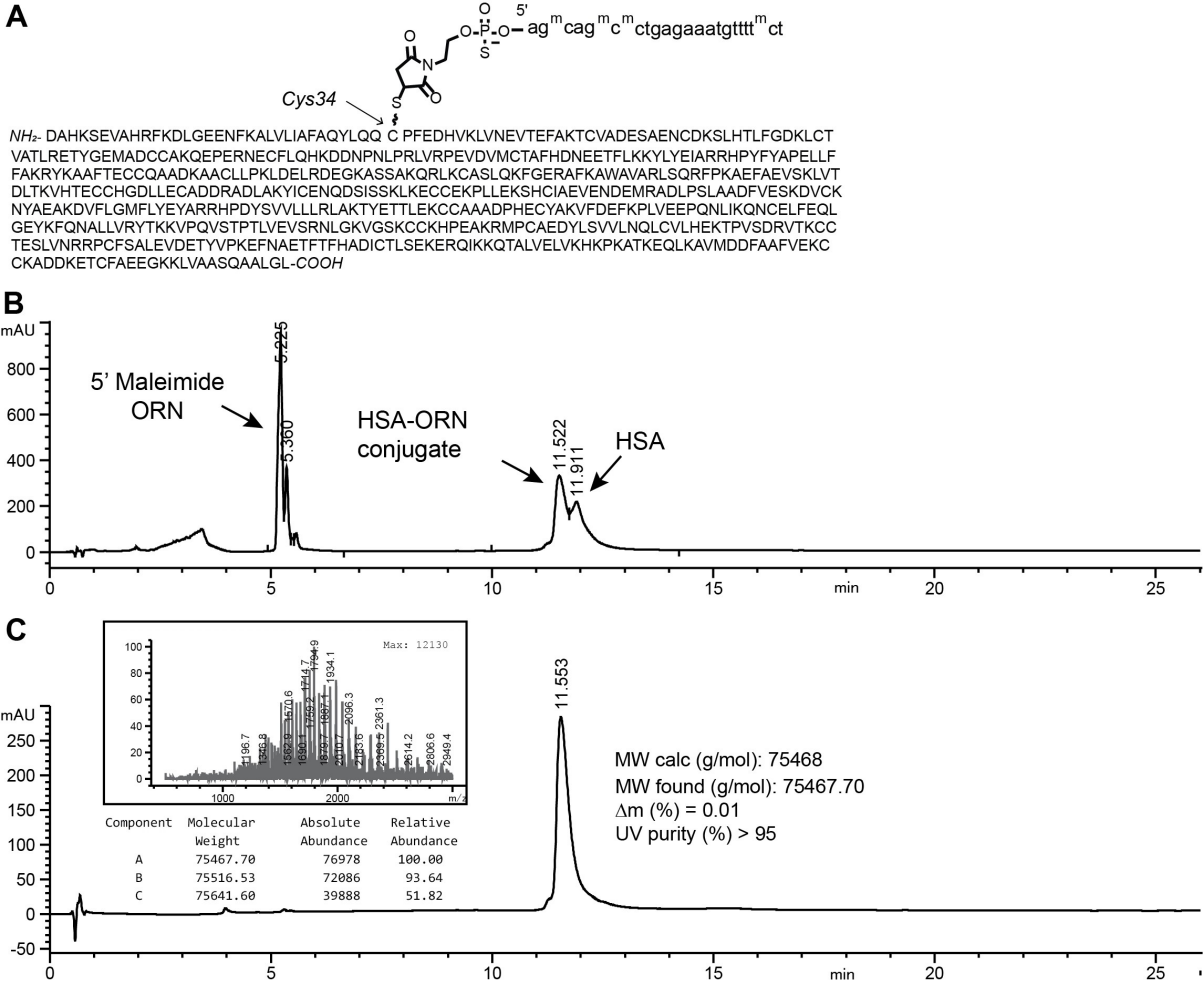
*Synthesis of a high‐purity 2’‐O‐MOE albumin oligonucleotide conjugate*. **A** Sequence and calculated molecular weight of the HSA‐oligonucleotide conjugate. ^m^C: 5‐methyl 2’‐*O*‐methoxyethyl cytosine. **B** LC‐MS chromatogram of the conjugate prior to RP‐HPLC. ORN: oligonucleotide; HSA: human serum albumin. **C** LC‐MS of the purified conjugate.

In summary, in this study we provide a robust methodology for the synthesis of 2’‐MOE PS cationic peptide conjugates. These bifunctional molecules are generally considered difficult to synthesize which, at least partly explains their lack of development to date. Indeed, most advanced CPPs are weakly charged^[7a]^ or combined with PMO chemistry. A significant finding of the work is that highly cationic CPPs can be conjugated to 2’‐MOE PS oligonucleotides and isolated in good yields, provided that denaturing conditions are used for conjugation and purification steps. We also synthesized non‐cationic, hydrophobic CPP conjugates (**25**–**26**). These peptides have been less thoroughly studied but have favorable uptake properties,^[30]^ which warrants investigation of their ability to deliver oligonucleotides *in vivo*. Importantly, and in prevision of biological applications, we confirmed for several conjugates the absence of self‐assembly into nanoparticles (Figure S4) and that peptide conjugation does not interfere with hybridization to an RNA target (Figure S5). A natural extension of this work will be to screen the library in a systematic fashion *in vitro* and determine whether correlations are obtained between hydrophobicity and spontaneous uptake into cells.

## Conflict of interest

The authors declare no conflict of interest.

## Supporting information

As a service to our authors and readers, this journal provides supporting information supplied by the authors. Such materials are peer reviewed and may be re‐organized for online delivery, but are not copy‐edited or typeset. Technical support issues arising from supporting information (other than missing files) should be addressed to the authors.

Supporting Information
