# Supplementary material for: Efficient Synthesis of 2’‐O‐Methoxyethyl Oligonucleotide‐Cationic Peptide Conjugates
Source: ChemMedChem. 2021 Sep 8;16(22):3391–5. doi: 10.1002/cmdc.202100388 (PMC9291120; doi:10.1002/cmdc.202100388)
Supplement: Supplementary file 1 — Supporting Information [file CMDC-16-3391-s001.pdf]

ChemMedChem

Supporting Information

Efficient Synthesis of 2′-*O*-Methoxyethyl Oligonucleotide-Cationic Peptide Conjugates

François Halloy, Alyssa C. Hill, and Jonathan Hall*

Table of contents

Methods 2
Oligonucleotide synthesis 2
5'-capped maleimide Intermediate (1) purification 2
Generation of the 5'-free maleimide Intermediate (2) 2
Peptide reagents 2
Peptide oligonucleotide conjugation 2
Purification of peptide oligonucleotide conjugates by IE-HPLC 2
Purification of peptide oligonucleotide conjugates by RP-HPLC 3
Human serum albumin oligonucleotide conjugation and purification 3
Yield determination for peptide oligonucleotide conjugates 3
LC-MS analytics 3
Dynamic light scattering (DLS) 3
Thermal melting 3
Figure S1. *Optimization of maleimide deprotection* 5
Figure S2. *IE-HPLC optimization for peptide-oligonucleotide conjugate purification* 6
Figure S3: *Retention times of selected conjugates on a C18 column.* 7
Figure S4: *DLS measurements for selected conjugates.* 7
Figure S5: *Thermal melting measurements for selected conjugates.* 8
Conjugate final chromatograms - Figures S6 to S30 9

Methods

Oligonucleotide synthesis

Oligonucleotide synthesis was conducted on a MerMade 12 synthesizer (BioAutomation Inc) on a 50 nmole scale. 500 Å UnyLinker controlled pore glass (CPG) solid support (ChemGenes) was used. Cleavage of the dimethoxytrityl group was carried out with a solution of 3% dichloroacetic acid v/v in dichloromethane. 2'-O-(2-methoxyethyl) (MOE) phosphoramidites (ThermoFisher Scientific) were prepared at 0.08 M concentration in dry acetonitrile. 5-(Benzylthio)-1H-tetrazole (Carbosynth) was used at 0.24 M concentration in dry acetonitrile to activate the phosphoramidites for coupling. Sulfurization was carried out with a 0.05 M solution of 3-((N,N-dimethylaminomethylidene)amino)-3H-1,2,4-dithiazole-5-thione (Glen Resarch) in a 1:1 v/v mixture of pyridine/acetonitrile. Capping of failed sequences was achieved with acetic anhydride in tetrahydrofuran (THF) and 16% N-methylimidazole in THF. The following cycle parameters were used: deblock 2 x 60 s; coupling 2 x 180 s; sulfurization 1 x 150 s; capping 2 x 50 s. 5'-maleimide modifier was purchased from Glen Research (#10-1938), dissolved at 0.1 M in dry acetonitrile and coupled according to the manufacturer's instruction. Upon synthesis completion, CPG was suspended in fresh 25 % ammonia (Sigma Aldrich) and shaken at 35°C for 16 hours. CPG was filtered and washed with 50 % aqueous ethanol (EtOH). Liquid fractions were pooled and evaporated to dryness to afford Intermediate (I), which was dissolved in RNAse-free water for HPLC purification.

5'-capped maleimide Intermediate (1) purification

Intermediate (1) was purified by RP-HPLC (Agilent) on a XBridge OST C18 reverse phase column with a concentration gradient of acetonitrile in 0.1 M aqueous triethylammonium acetate buffer, pH 8. Product-containing fractions were pooled and evaporated to dryness. Purity was analyzed by LC-MS (see below) and aliquoted for long term storage at -20°C.

Generation of the 5'-free maleimide Intermediate (2)

Uncapping of Intermediate (1) was performed immediately before peptide conjugation. Intermediate (1) was microwave-irradiated in water for 90 minutes at 90°C. The reaction mixture was immediately vacuum-concentrated in a Speedvac, until 70-80% volume reduction was achieved (no evaporation to dryness). Completion of the retro Diels-Alder reaction was verified by LC-MS injection. Reaction mixtures with >80% conversion into Intermediate (2) were used for peptide conjugation.

Peptide reagents

Peptides were purchased from GenScript (NJ, United States) and were more than 80 % pure according to HPLC. Peptides were stored lyophilized at -20°C. Working aliquots were prepared directly prior to use.

Peptide oligonucleotide conjugation

Several conjugation buffers were used in the study. Buffer A: 0.1 M K_2HPO_4 pH 6.8, 0.3 M KBr, 8 M urea; Buffer B: 70% v/v formamide, 0.2 M triethylammonium acetate (TEAA) pH 6.8; Buffer C: 50 % DMA, 30 mM KBr, 0.8 M urea, 10 mM K_2HPO_4 pH 6.8; Buffer D: 20% DMA, 0.33 M TEAA pH 8. Buffer A was used for most conjugations excepted with Penetratin (Buffer C) or human serum albumin (Buffer D; see below). Buffer B was used in preliminary experiments only (see **Figure S2**).

Intermediate (II) was first prepared in the buffer of choice. Six molar equivalents of peptide were added. Oligonucleotide concentration was 50 µM; final peptide concentration was 300 µM. All reactions were run for 1 hour at room temperature, with gentle shaking on a thermomixer. LC-MS injections were performed to control the conjugation rate. To reactional mixtures with less than 50% conversion, a further 2.5 eq of peptide was added and conjugation was allowed to run for one more hour (3 hours in the case of Penetratin). Conjugation of the H5WYG peptide in Buffer A required microwave irradiation, which was conducted at 1 W for 30 min in a CEM reactor. All conjugates were purified by IE-HPLC or RP-HPLC as detailed below.

Purification of peptide oligonucleotide conjugates by IE-HPLC

Peptide oligonucleotide conjugates were purified by IE-HPLC (Agilent) on a Resource Q column (GE Healthcare) at 1 ml/min and 35°C. Buffer 1: 50 mM K_2HPO_4 pH 6.8, 5 M urea, 30% v/v ACN. Buffer 2: 50 mM K_2HPO_4 pH 6.8, 1.2 M KBr, 5 M urea, 30% v/v ACN. Gradient: 0 to 50 % Buffer 2. Pure fractions were pooled and immediately desalted by three rounds of ultracentrifugation in nuclease-free water (4

ml Amicon columns, 3 kDa cut-off, Merck). Concentrates were transferred into a screw-cap tube. Conjugates were quantified by Nanodrop measurement and their purity evaluated by LC-MS injection. Conjugates were kept at -20°C for long-term storage.

Purification of peptide oligonucleotide conjugates by RP-HPLC

Conjugates were purified by RP-HPLC (Agilent) on a XBridge OST C18 reverse phase column (4 ml/min, 40°C) and a concentration gradient of acetonitrile in 0.1 M aqueous triethylammonium acetate buffer, pH 6.8. Pure fractions were concentrated in a Speedvac prior to redissolution in Nanopure water. Conjugates were quantified by Nanodrop measurement and their purity evaluated by LC-MS injection. Conjugates were kept at -20°C for long-term storage.

Human serum albumin oligonucleotide conjugation and purification

A modified conjugation protocol was used for conjugation to human serum albumin. First, 5 molar equivalents of albumin (Sigma-Aldrich #A3782) were dissolved in Buffer D (20% DMA, 0.33 M TEAA pH 8). The oligonucleotide was added at a final concentration of 300 µM. Conjugation was allowed to run for 1.5 hours prior to RP-HPLC purification with a XBridge OST C18 reverse phase column (5 ml/min, 60°C) and a concentration gradient of acetonitrile in 0.1 M TEAA, pH 6.8. Pure fractions were pooled and concentrated in a Speedvac. The soluble fraction was collected, quantified by Nanodrop measurement, and its purity evaluated by LC-MS injection. The conjugate was kept at -20°C for long-term storage.

Yield determination for peptide oligonucleotide conjugates

The molar extinction coefficient at 260 nm of the conjugate $\varepsilon_{260,\ conjugate}$ was determined as the sum of the molar extinction coefficients of the peptide ($\varepsilon_{260,\ peptide}$) and oligonucleotide ($\varepsilon_{260,\ oligonucleotide}$) moieties. $\varepsilon_{260,oligonucleotide}$ was calculated from an online tool[1] as 266880 $M^{-1}cm^{-1}$. $\varepsilon_{260,\ peptide}$ was calculated according to the following equation: $\varepsilon_{260,\ peptide} = (nW\times4000) + (nY\times820) + (nF\times130) + (nC\times120)\ M^{-1}cm^{-1}$, where nW, nY, nF, nC correspond to the number of tryptophan, tyrosine, phenylalanine and cysteine in the peptide and 4000, 820, 130 are their respective molecular extinction coefficients. Calculated $\varepsilon_{260,\ conjugate}$ and $\varepsilon_{260,\ peptide}$ values are shown in **Table S1**.

LC-MS analytics

Oligonucleotide conjugates were analyzed by LC-MS (Agilent 1200/6130 system) on a Waters Acquity OST C-18 column, 2.1 x 50 mm, 1.7µm, 65°C. Buffer A: 0.4 M hexafluoroisopropanol (HFIP), 15 mM triethylamine; Buffer B: methanol. Gradient: 10 - 50% buffer B; flow rate: 0.3 $ml.min^{-1}$; temperature 65°C.

Dynamic light scattering (DLS)

Samples were prepared at 2 µM in 100 µL ultrapure water and analyzed for size by means of DLS using a Zetasizer Pro (Malvern Panalytical) at 25°C. Each experiment was performed three times.

Thermal melting

Samples were prepared at 2 µM in 200 µL 100 mM NaCl, 10 mM phosphate buffer, 0.1 mM Na_2EDTA, pH 7.0. Absorbance at 260 nm was measured over the temperature range 20°C to 90°C on a CARY 300 spectrophotometer (Varian, Inc.). Each temperature series was performed three times. We used hold periods of 5 min at both 5°C and 90°C to ensure thermal equilibrium. The heating rate was 0.5°C/min, and the data collection interval was 0.5°C. Mean melting temperatures (T_ms) were determined from first derivative analyses of nonlinear fit melting curves in Microsoft Excel.

[1] OligoCalc version 3.27, http://biotools.nubic.northwestern.edu/OligoCalc.html

Table S1. *Calculated peptide and conjugate molar extinction coefficients*

Peptide sequence (N→C)	ε 260, peptide ($M^{1}cm^{-1}$)	ε 260, oligonucleotide ($M^{-1}cm^{-1}$)	ε 260,conjugate ($M^{-1}cm^{-1}$)
CRKKRRQRRRPPQ	120	266880	267000
CRQIKIWFQNRRMKWKKGG	8250		275130
CRRRRRRRQIKIWFQNRRMKWKKGG	8250		275130
CGLFHAIAHFIHGGWHGLIHGWYG	9200		276080
CRRRRRRRRRFF	380		267260
CHAIYPRH	940		267820
CTHRPPMWSPVWP	8120		275000
CVQRKRQKLMP	120		267000
CSKKKKTKV	120		267000
CPKKKRKV	120		267000
CQEQLERALNSS	120		267000
CESGGGGSPGRRRRRRRRRRR	120		267000
CNDTIPEDFQEFQTQNFDRFDN	640		267520
CSTFTKSP	250		267130

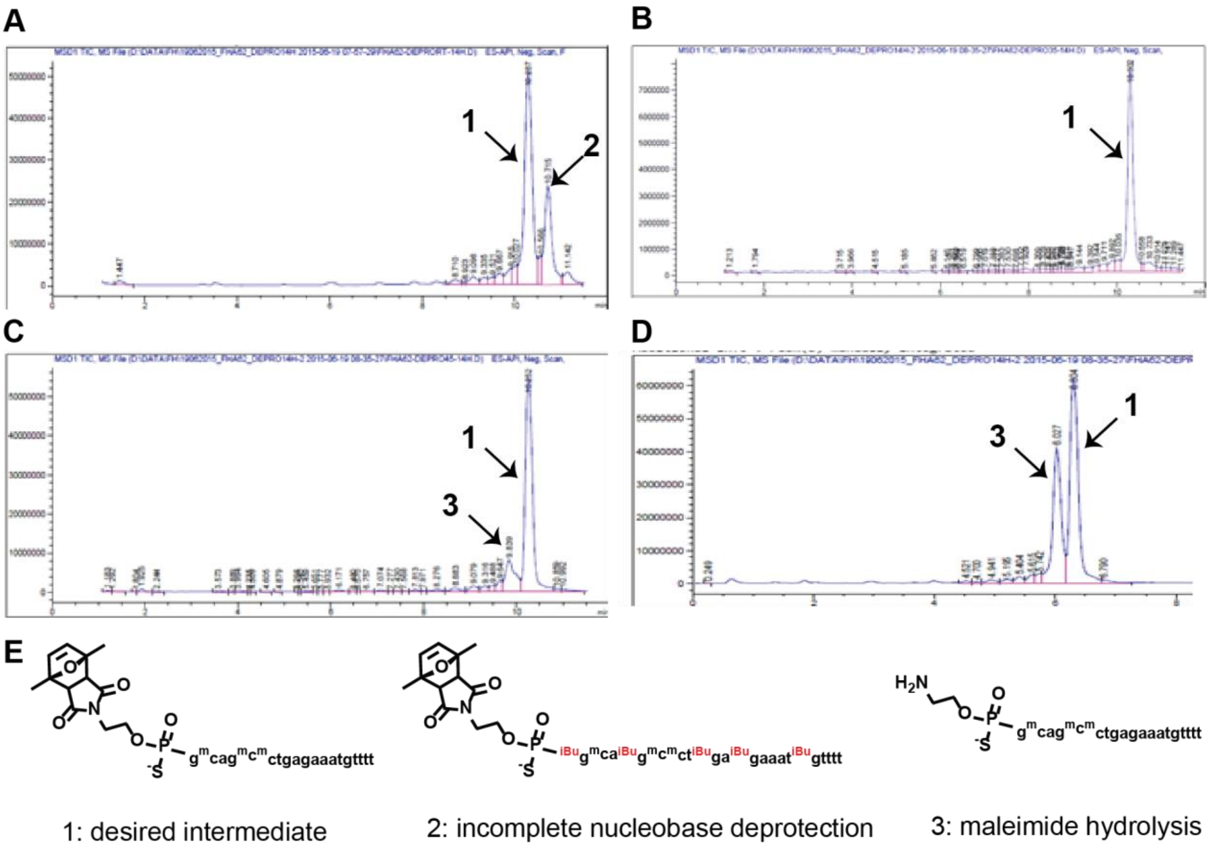

Figure S1. *Optimization of maleimide deprotection*

The 5’-capped maleimide oligonucleotide on solid support was deprotected in 25% aqueous ammonia. The reaction was run overnight at room temperature (**A**), 35°C (**B**), 45°C (**C**), or 50°C (**D**). Volatiles were briefly evaporated before LC-MS injection. Incomplete nucleobase deprotection was observed at room temperature (product 2), with M+71 adducts likely arising from isobutyryl groups not removed from G nucleobases. Alkaline hydrolysis of the maleimide was observed at 45°C and 50°C (product 3). Deprotection at 35°C (**B**) yielded the desired intermediate in good purity and ready for RP-HPLC purification. ^{m}C: 5-methyl 2’-O-methoxyethyl cytosine ; ^{iBu}G: 2’-O-methoxyethyl N-isobutyryl guanosine.

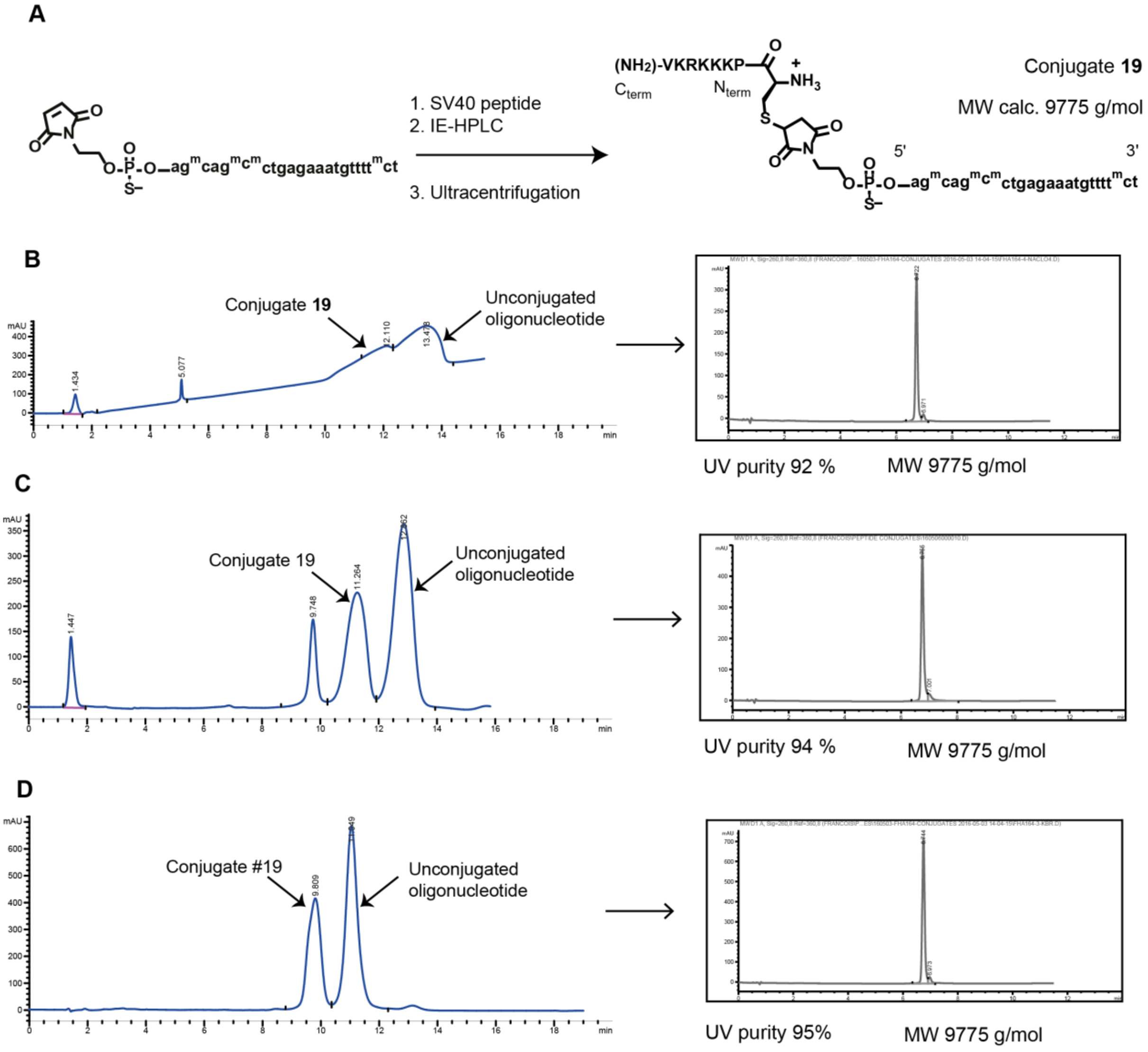

Figure S2. *IE-HPLC optimization for peptide-oligonucleotide conjugate purification*

A The SV40 nuclear-localization peptide conjugate **19** was elected for IE-HPLC optimization (see **Table 1**). Conjugation was performed in a buffer of K_2HPO_4/KBr/urea (Buffer A) or in 70 % formamide/TEAA pH 6.8 (Buffer B) as detailed in the Methods section.

IE-HPLC was performed from Buffer B with concentration gradient of aq. NaClO4 (**B**). *Buffer 1:* 20 mM Tris-HCl pH 6.8, 50 % formamide, 1 mM TCEP; *Buffer 2:* 20 mM Tris-HCl pH 6.8, 50 % formamide, 1 mM TCEP, 0.4 M $NaClO_4$; *Gradient* 0 to 100% Buffer 2.

Alternatively, IE-HPLC was performed from Buffer B with a concentration gradient of NaCl (**C**). *Buffer 1:* 50 mM K_2HPO_4 pH 6.8, 50 % formamide, 1 mM TCEP; *Buffer 2:* 50 mM K_2HPO_4 pH 6.8, 50 % formamide, 1 mM TCEP, 1 M NaCl; *Gradient* 0 to 100% Buffer 2

D IE-HPLC was performed from Buffer A with a concentration gradient of aq. KBr. *Buffer 1:* 50 mM K_2HPO_4 pH 6.8, 5 M urea, 30 % acetonitrile, 1 mM TCEP; *Buffer 2:* 50 mM K_2HPO_4 pH 6.8, 5 M urea, 30 % acetonitrile, 1 mM TCEP, 1.2 M KBr; *Gradient* 0 to 60% Buffer 2.

(**B**-**D**) Final LC-MS analytics are given at the right-hand-side for each system.

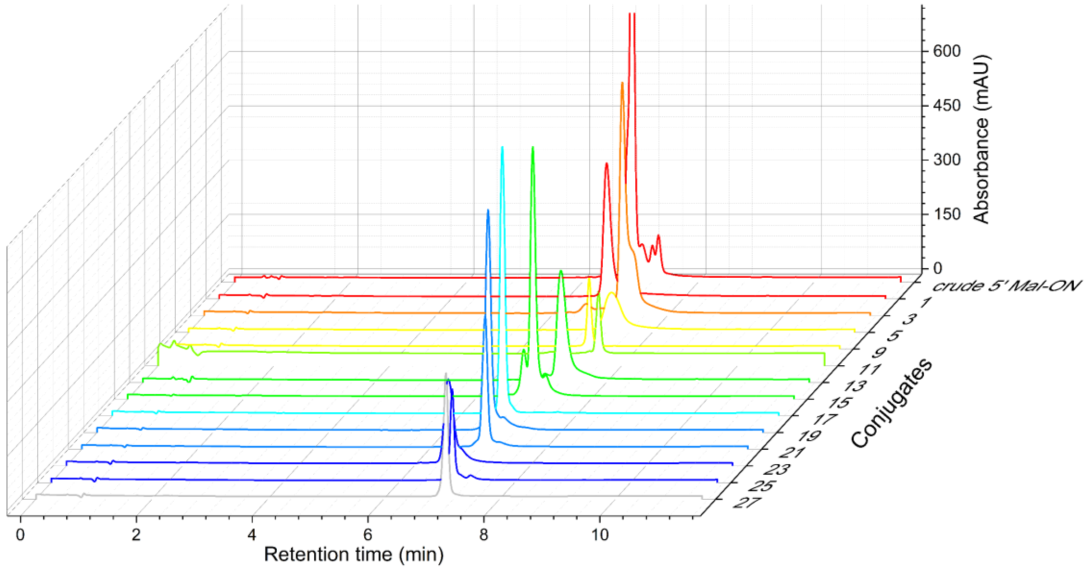

Figure S3: *Retention times of selected conjugates on a C18 column.*
5' free maleimide Intermediate (II) and selected peptide conjugates (odd entries in **Table 1**) were injected in LC-MS for comparison of their retention times on a reverse phase column. A 10 to 50% concentration gradient of methanol in 0.4 M HFIP, 15 mM triethylamine was used on a Waters Acquity OST C18 analytical column.

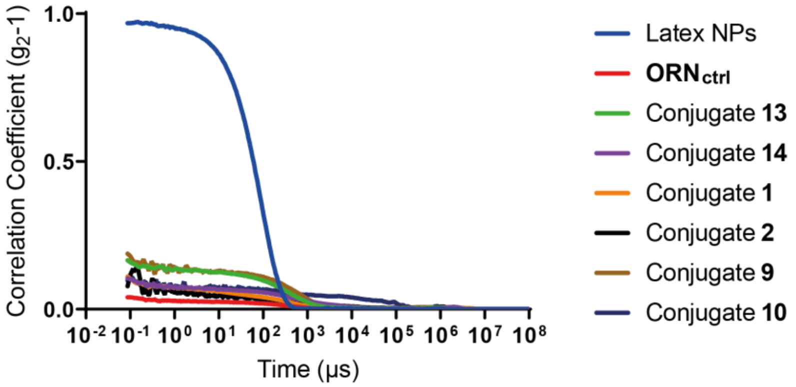

	Derived Mean Count Rate (kcps)	Standard deviation
Latex NPs	30430.0	19.47
**ORN**$_{\mathbf{ctrl}}$	390.4	16.77
Conjugate **13**	219.7	37.17
Conjugate **14**	195.5	72.42
Conjugate **1**	185.3	25.01
Conjugate **2**	91.53	N/A[1]
Conjugate **9**	89.27	11.24
Conjugate **10**	115	N/A[1]

Figure S4: *DLS measurements for selected conjugates.*
Oligonucleotide samples were prepared at 2 µM in ultrapure water in a final volume of 100 µl and analyzed for size by means of DLS using a Zetasizer Pro (Malvern Panalytical) at 25°C. Latex nanoparticles (NPs) were used as positive control. **ORN**$_{\mathbf{ctrl}}$ is the unconjugated oligonucleotide control.
[1] indicates n = 1 measurement only.

A

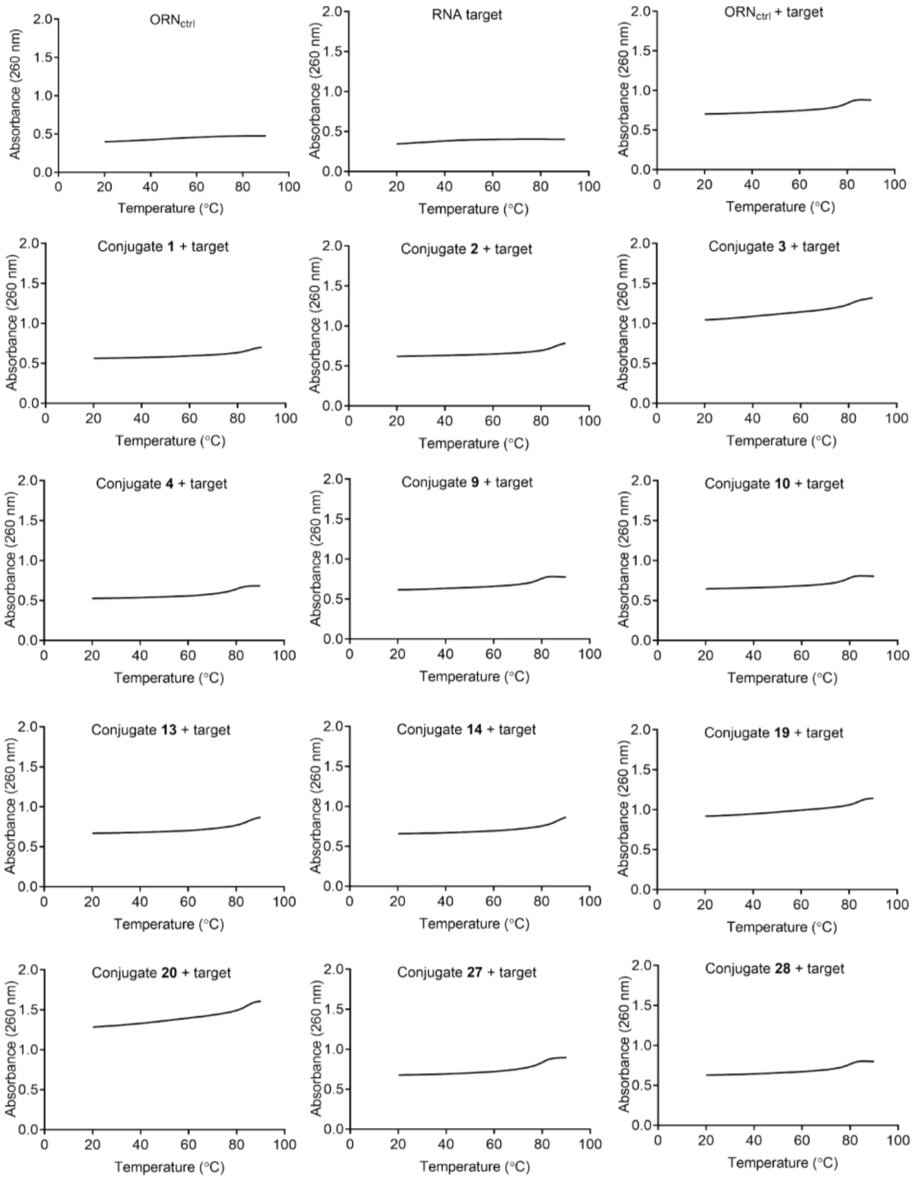

B

Conjugate	Peptide charge (pH 7)	T_m (°C)
**1**	9	> 80
**2**	7	> 80
**3**	8	ND[†]
**4**	6	78.2
**9**	1	78.1
**10**	1	77.9
**13**	9	> 80
**14**	9	> 80
**19**	6	> 80
**20**	4	> 80
**27**	1	78.3
**28**	1	78.7
**ORN_{ctrl}**	-	78.9

Figure S5: *Thermal melting measurements for selected conjugates.*
A Melting curve profiles of the unconjugated oligonucleotide **ORN_{ctrl}** and a selection of conjugates. A 22-nt RNA reverse complementary strand was used for this experiment. **B** Mean melting temperatures (T_ms) were calculated from first derivative analyses of nonlinear fit melting curves. Cationic peptide conjugates (**1, 2, 13, 14, 19, 20**) had increased T_m values, for which the upper baseline was too short for the fitting method. For these conjugates, T_m*s* were estimated at 85°C based on the maxima of first derivative plots. Conjugates **9, 10, 27, 28** (weakly charged peptides) had T_m*s* similar to the parent oligonucleotide **ORN_{ctrl}**. [†]poor fit; estimated T_m was 80°C.

Conjugate final chromatograms - Figures S6 to S30

Conjugates were run with a 10 to 50% concentration gradient of methanol in 0.4 M HFIP, 15 mM triethylamine on a Waters Acquity OST C18 analytical column. MW: molecular weight.

LCMS chromatograms of conjugates **1** and **5** are presented in **Figure 2**.

Figure S6. Conjugate **2**

MW (g/mol): calculated 10797.15, found 10797.31; Δm (%) = 0.01

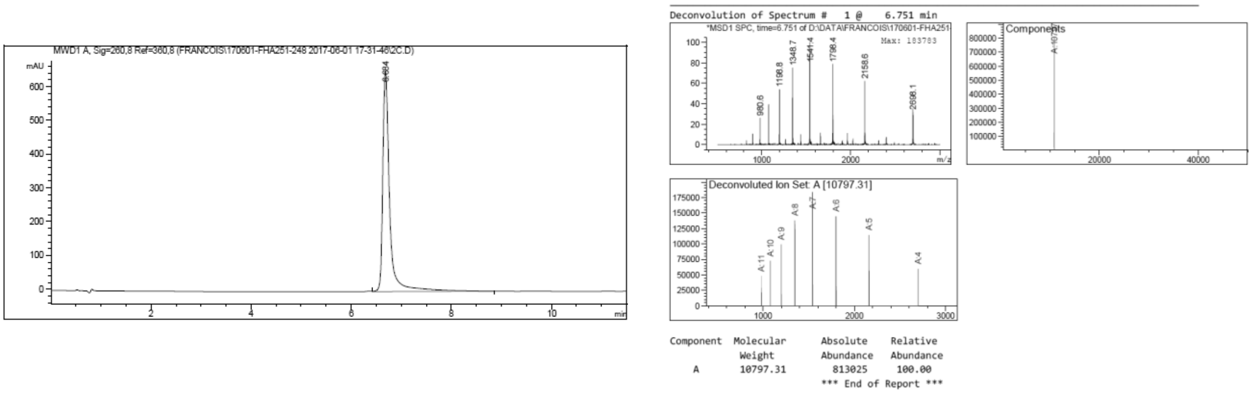

Figure S7. Conjugate **3**

MW (g/mol): calculated 11452.7, found 11453.42, 11481.26; Δm (%) = 0.01

The shoulder peak is an uncharacterized impurity.

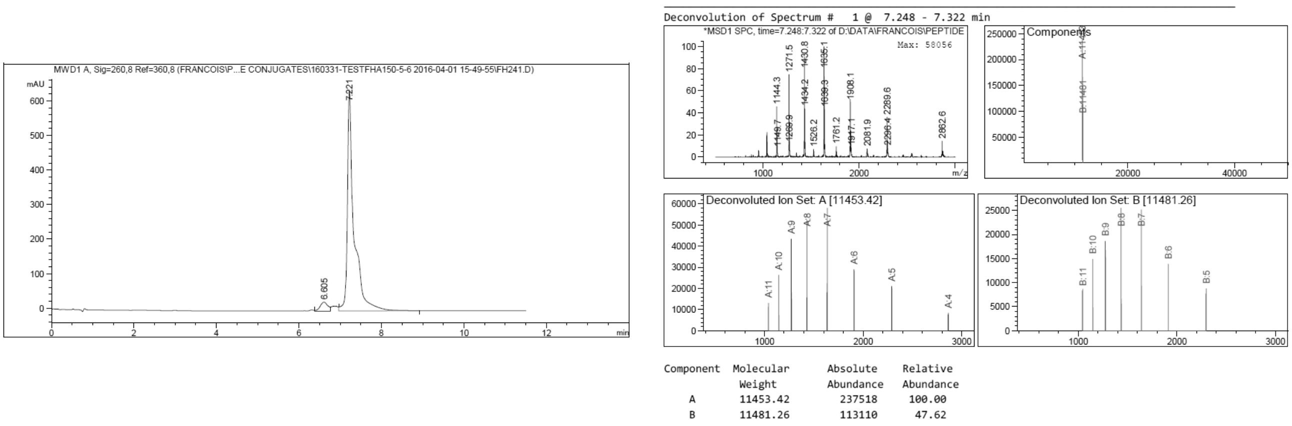

Figure S8. Conjugate **4**

MW(g/mol): Calculated 11496.0, found 11496.4; Δm (%) <0.01

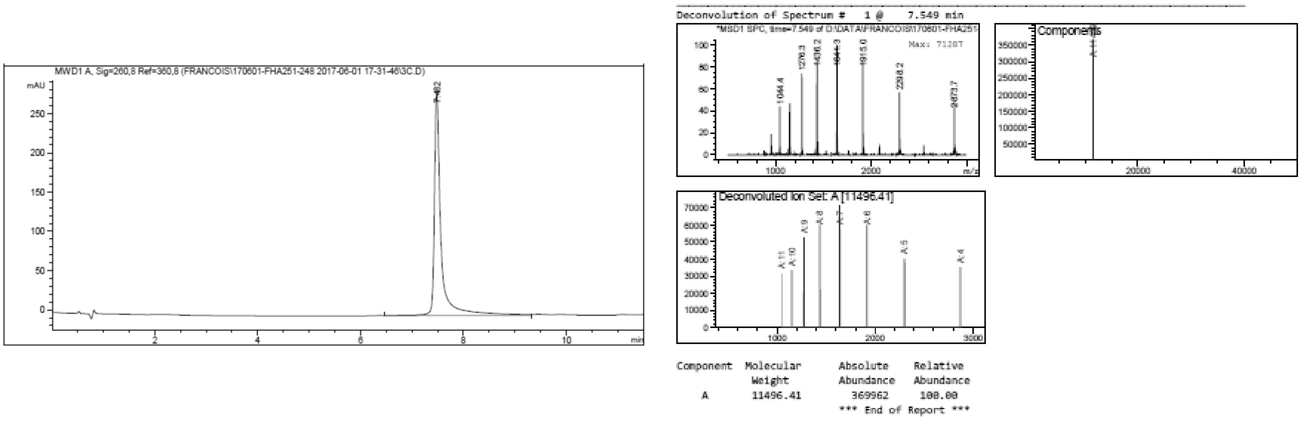

Figure S9. Conjugate **6**
MW(g/mol): Calculated 12391.1, found 12390.8; Δm (%) <0.01

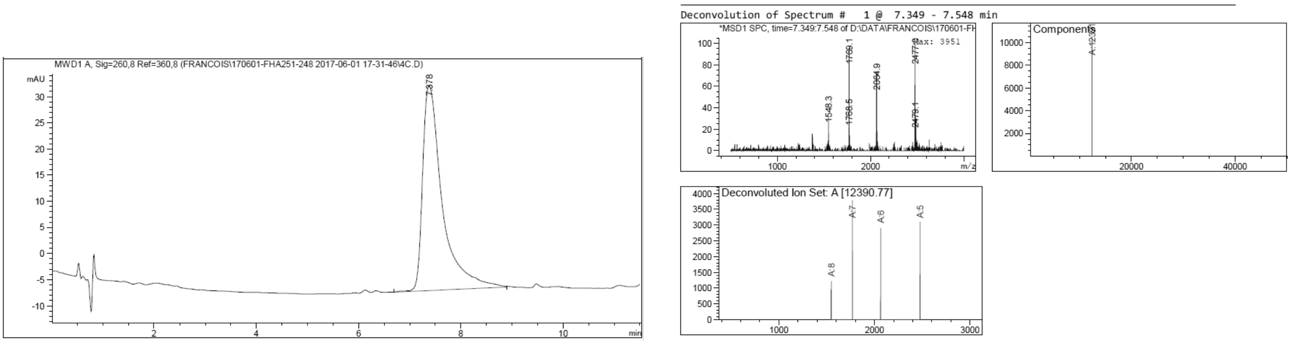

Component	Molecular Weight	Absolute Abundance	Relative Abundance
A	12390.77	10501	100.00

*** End of Report ***

Figure S10. Conjugate **7**
MW(g/mol): Calculated 11676.10, found 11676.38; Δm (%) <0.01

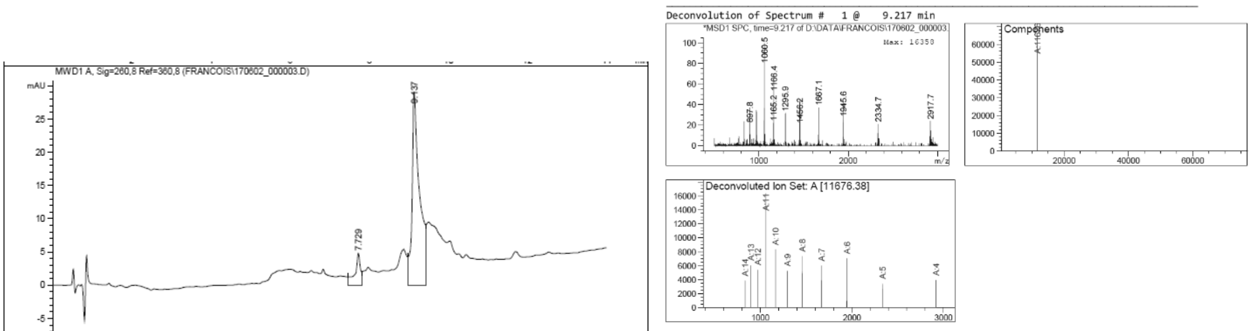

Component	Molecular Weight	Absolute Abundance	Relative Abundance
A	11676.38	64161	100.00

*** End of Report ***

Figure S11. Conjugate **9**
MW(g/mol): Calculated 9986.24, found 9986.15, 10028.86 (+K), 10044 (+acetate); Δm (%) <0.01

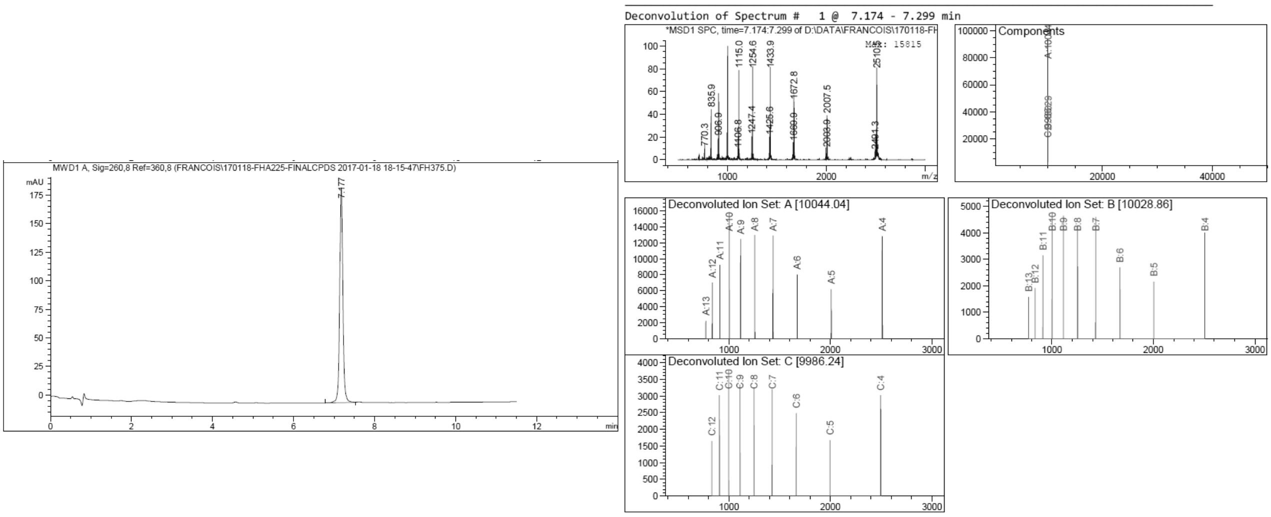

Component	Molecular Weight	Absolute Abundance	Relative Abundance
A	10044.04	93722	100.00
B	10028.86	31414	33.52
C	9986.24	24443	26.08

*** End of Report ***

<u>Figure S12.</u> Conjugate **10**

Mass (g/mol): Calculated 9986.24, found 10043.55 (+acetate), 10029.30 (+K); ∆m (%) = 0.57

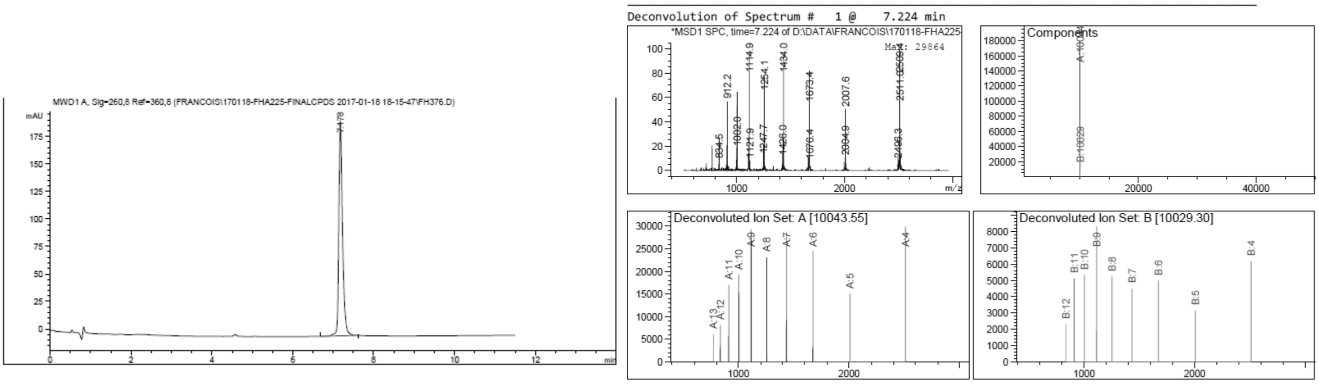

Component	Molecular Weight	Absolute Abundance	Relative Abundance
A	10043.55	178762	100.00
B	10029.30	33112	18.52

*** End of Report ***

<u>Figure S13.</u> Conjugate **11**

MW (g/mol): Calculated 10583.6, found 10584.17; ∆m (%) = 0.01

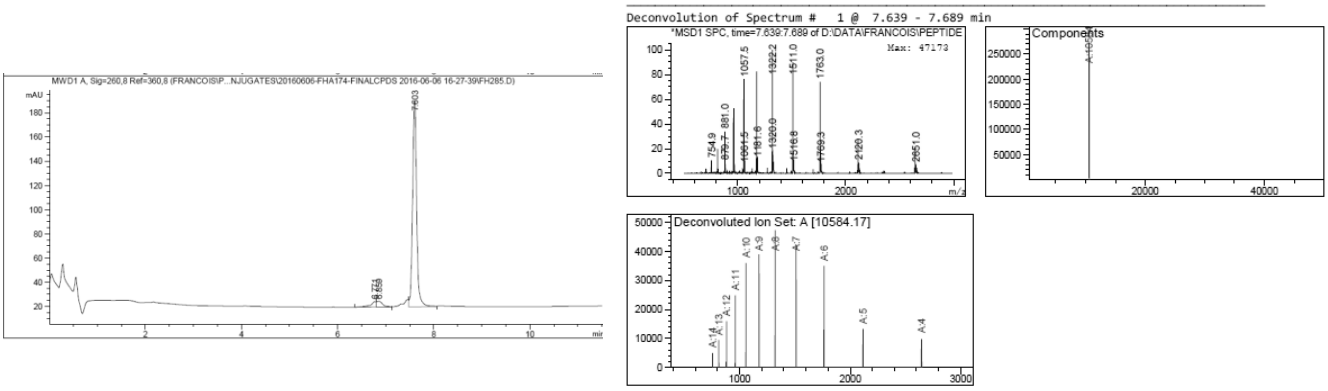

Component	Molecular Weight	Absolute Abundance	Relative Abundance
A	10584.17	273037	100.00

<u>Figure S14.</u> Conjugate **12**

MW (g/mol): Calculated 10583.6, found 10584.52, 10644.03 (acetate); ∆m (%) = 0.01

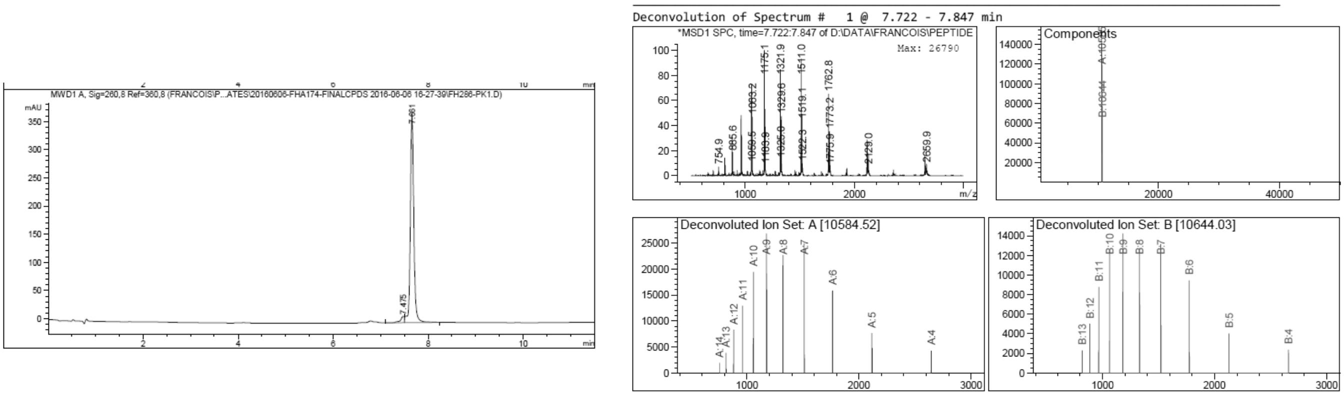

Component	Molecular Weight	Absolute Abundance	Relative Abundance
A	10584.52	141625	100.00
B	10644.03	76979	54.35

*** End of Report ***

Figure S15. Conjugate **13**
MW (g/mol): calculated 10811.19, found 10812.45, 10849.91 (+K); Δm (%) = 0.01

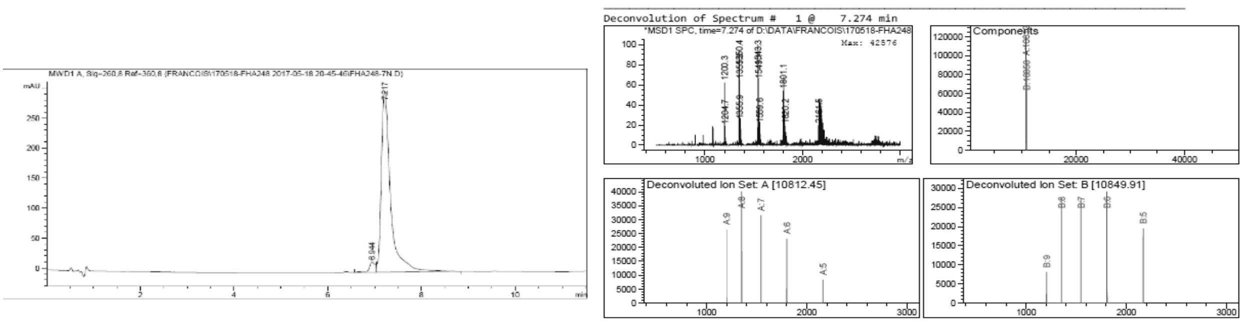

Component	Molecular Weight	Absolute Abundance	Relative Abundance
A	10812.45	117788	100.00
B	10849.91	95457	81.04

*** End of Report ***

Figure S16. Conjugate **14**
MW (g/mol): calculated 10811.19, found 10812.12; Δm (%) = 0.01

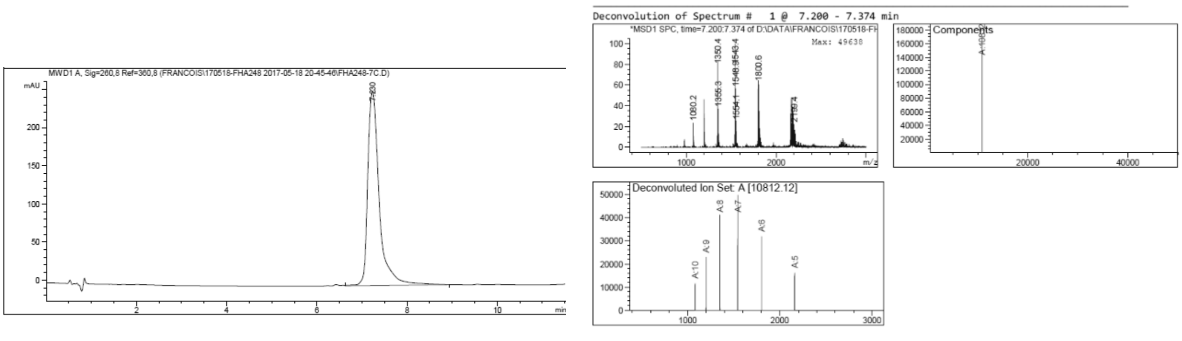

Component	Molecular Weight	Absolute Abundance	Relative Abundance
A	10812.12	169249	100.00

*** End of Report ***

Figure S17. Conjugate **15**
MW (g/mol): calculated 10375.52, found 10376.58, 10404.15, 10360.36; Δm (%) = 0.01

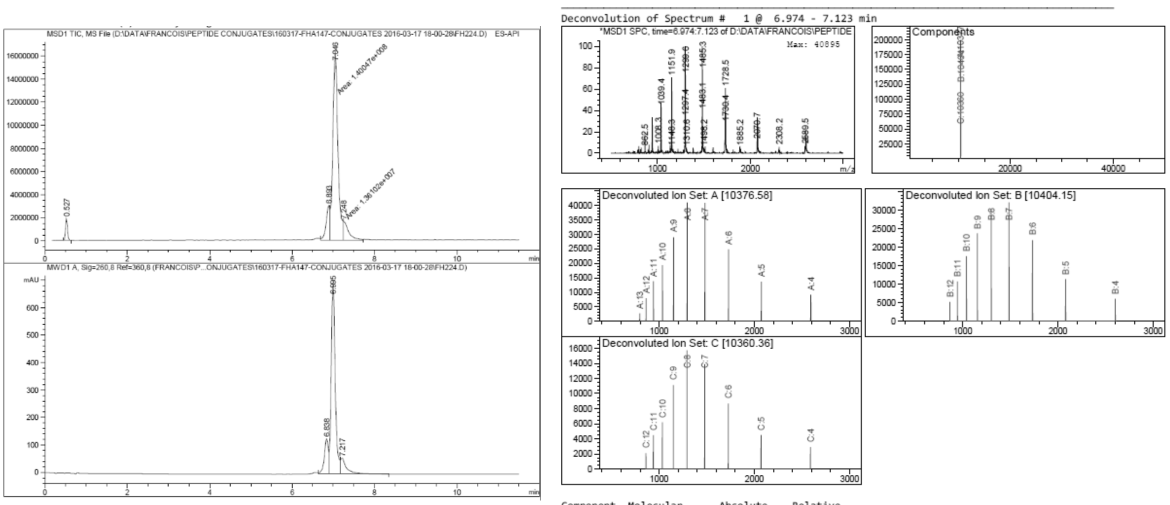

Component	Molecular Weight	Absolute Abundance	Relative Abundance
A	10376.58	199472	100.00
B	10404.15	151369	75.88
C	10360.36	68527	34.35

Figure S18. Conjugate **16**

MW(g/mol): calculated 10418.55, found 10419.12; Δm (%) = 0.01

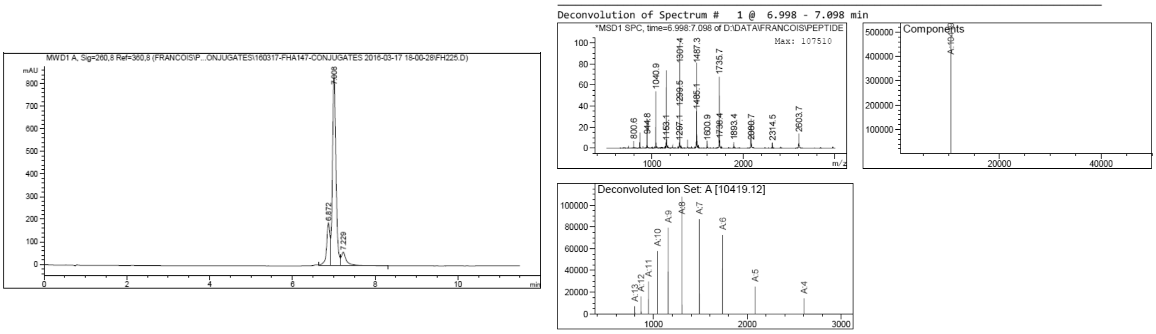

Component	Molecular Weight	Absolute Abundance	Relative Abundance
A	10419.12	482401	100.00

Figure S19. Conjugate **17**

MW(g/mol): calculated 10038.05, found 10038.57; Δm (%) = 0.01

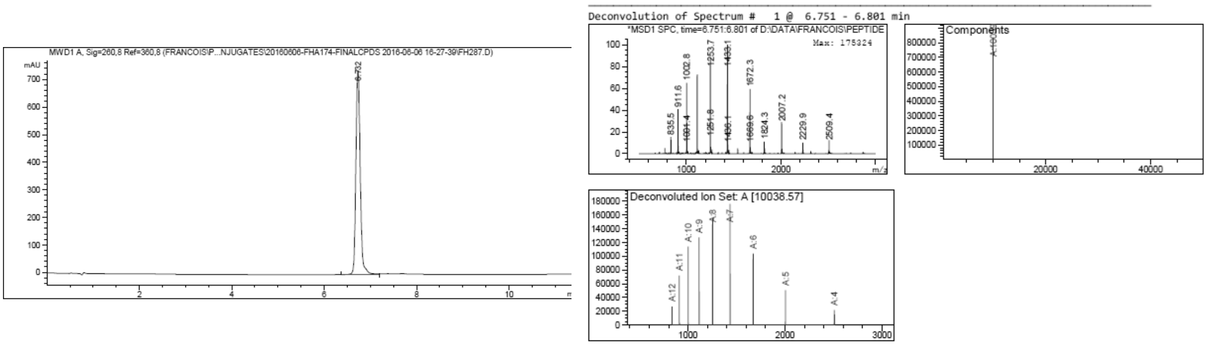

Component	Molecular Weight	Absolute Abundance	Relative Abundance
A	10038.57	831118	100.00

*** End of Report ***

Figure S20. Conjugate **18**

MW (g/mol): calculated 10081.07, found 10081.70; Δm (%) = 0.01

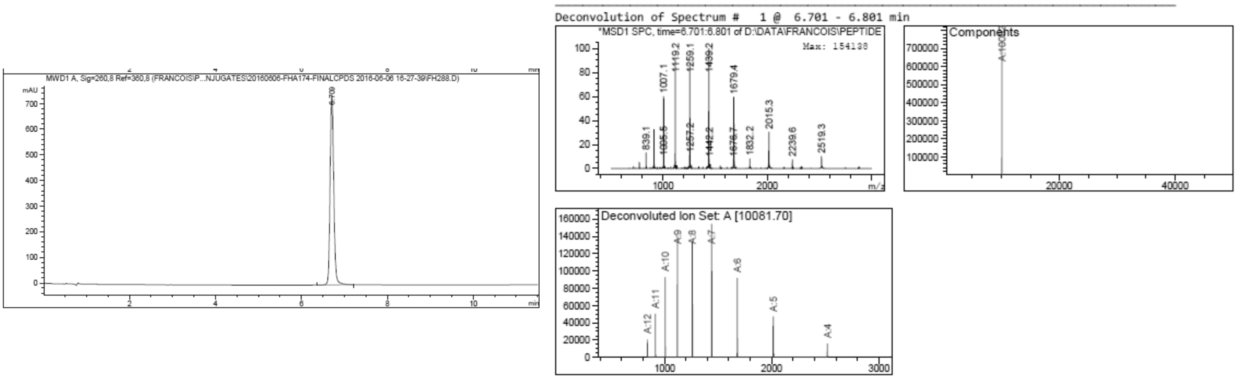

Component	Molecular Weight	Absolute Abundance	Relative Abundance
A	10081.70	739307	100.00

*** End of Report ***

Figure S21. Conjugate **19**
MW (g/mol): calculated 9975.07, found 9975.8; Δm (%) = 0.01

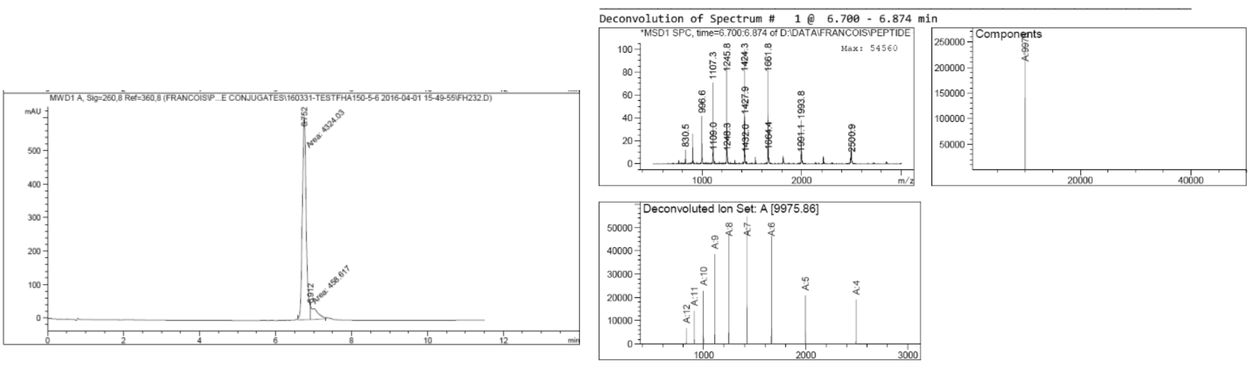

Component	Molecular Weight	Absolute Abundance	Relative Abundance
A	9975.86	248751	100.00

Figure S22. Conjugate **20**
MW(g/mol): calculated 10018.09, found 10018.91; Δm (%) = 0.01

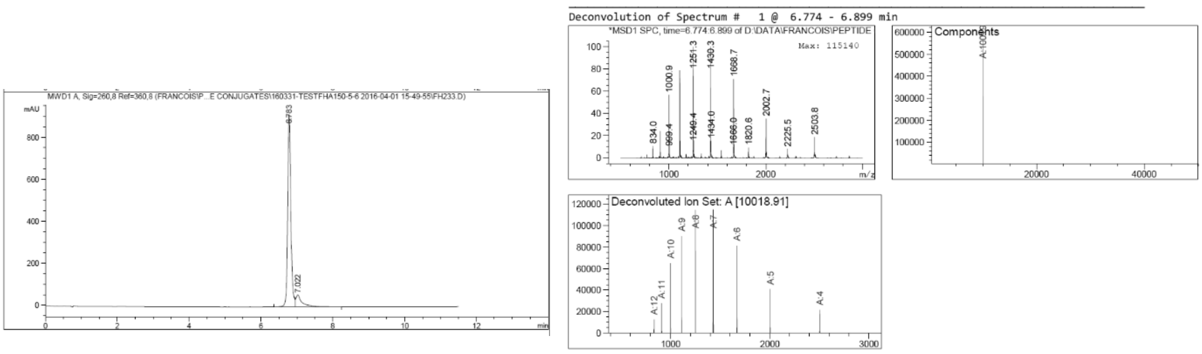

Component	Molecular Weight	Absolute Abundance	Relative Abundance
A	10018.91	563492	100.00

Figure S23. Conjugate **21**
MW(g/mol): calculated 10367.49, found 10368.32; Δm (%) = 0.01

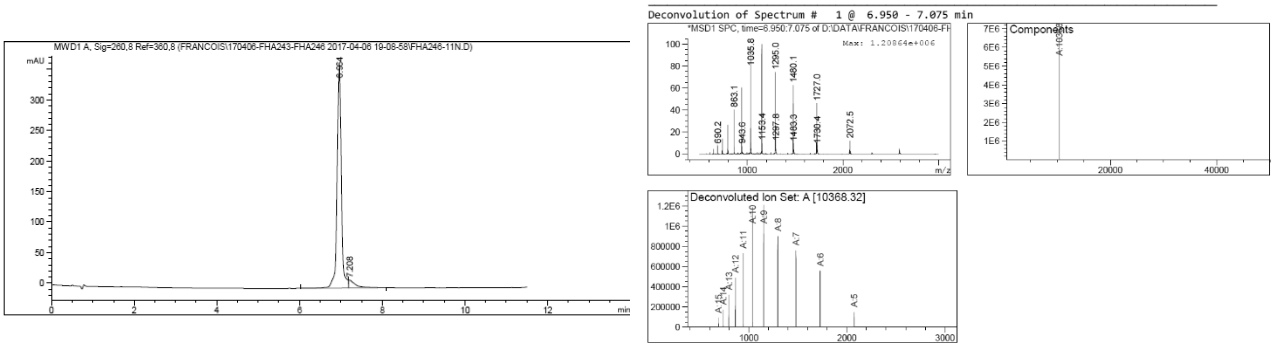

Component	Molecular Weight	Absolute Abundance	Relative Abundance
A	10368.32	6521980	100.00

Figure S24. Conjugate **22**
MW(g/mol): calculated 10367.49, found 10351.23; Δm (%) = 0.15

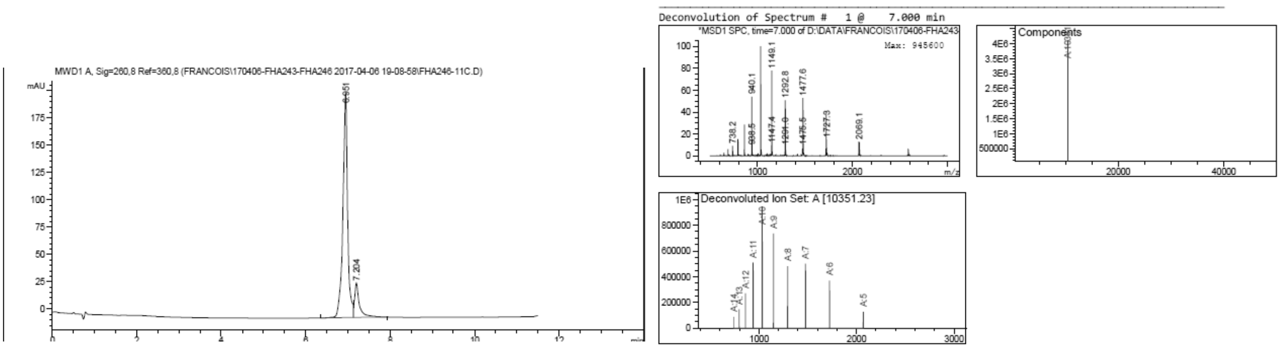

Component	Molecular Weight	Absolute Abundance	Relative Abundance
A	10351.23	4117095	100.00

Figure S25. Conjugate **23**
MW (g/mol): calculated 11514.55 found 11515.05; Δm (%) < 0.01

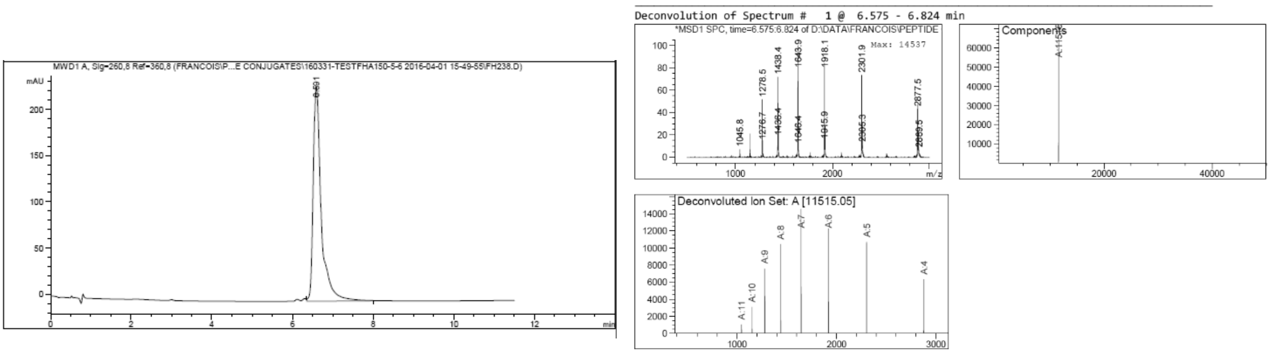

Component	Molecular Weight	Absolute Abundance	Relative Abundance
A	11515.05	64589	100.00

Figure S26. Conjugate **24**
MW (g/mol): calculated 11514.55 found 11514.43, 11554.47 (+K); Δm (%) < 0.01

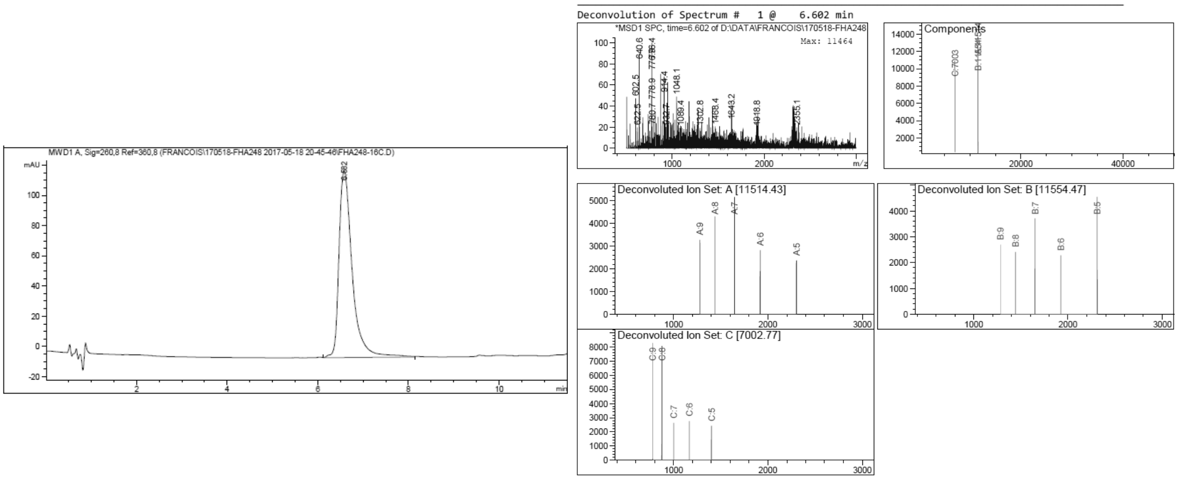

Component	Molecular Weight	Absolute Abundance	Relative Abundance
A	11514.43	13726	100.00
B	11554.47	11447	83.40
C	7002.77	10918	79.54

*** End of Report ***

Figure S27. Conjugate **25**
MW (g/mol): calculated 11713.79, found 11714.62, 11698.29; Δm (%) = 0.01

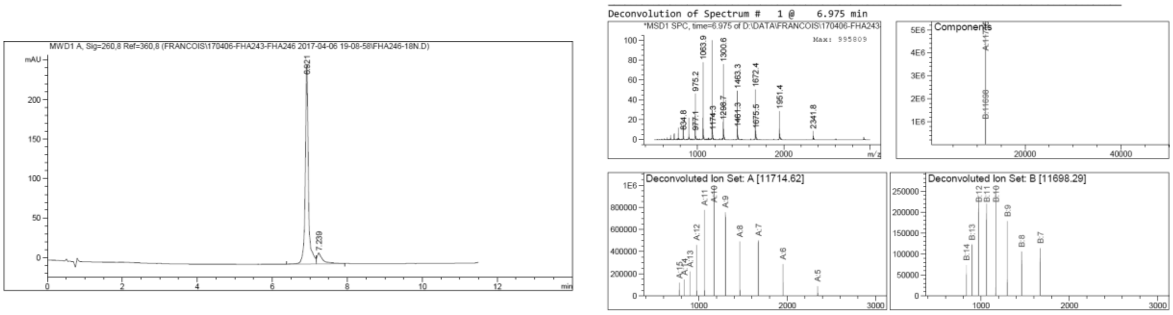

Component	Molecular Weight	Absolute Abundance	Relative Abundance
A	11714.62	4800847	100.00
B	11698.29	1289056	26.85

Figure S28. Conjugate **26**
MW (g/mol): calculated 11755.83, found 11756.67, 11794.50 (+K); Δm (%) = 0.01

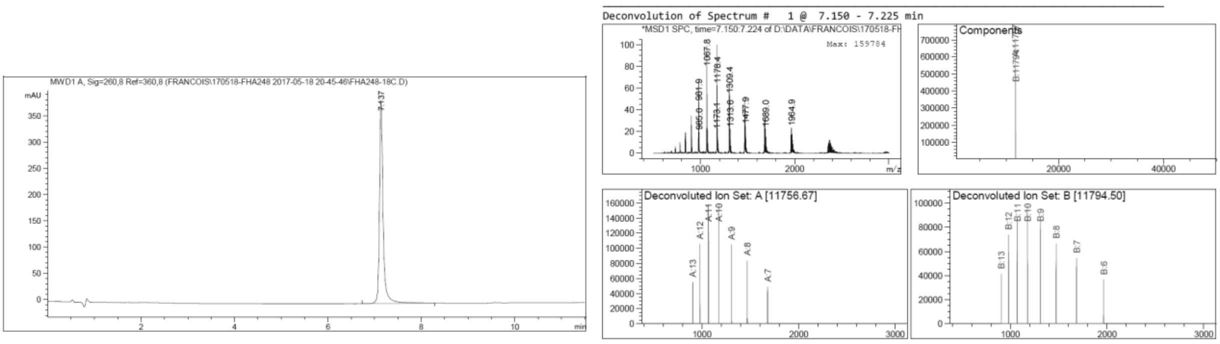

Component	Molecular Weight	Absolute Abundance	Relative Abundance
A	11756.67	707457	100.00
B	11794.50	532381	75.25

*** End of Report ***

Figure S29. Conjugate **27**
MW (g/mol): calculated 9859.15, found 9860.18; Δm (%) = 0.01

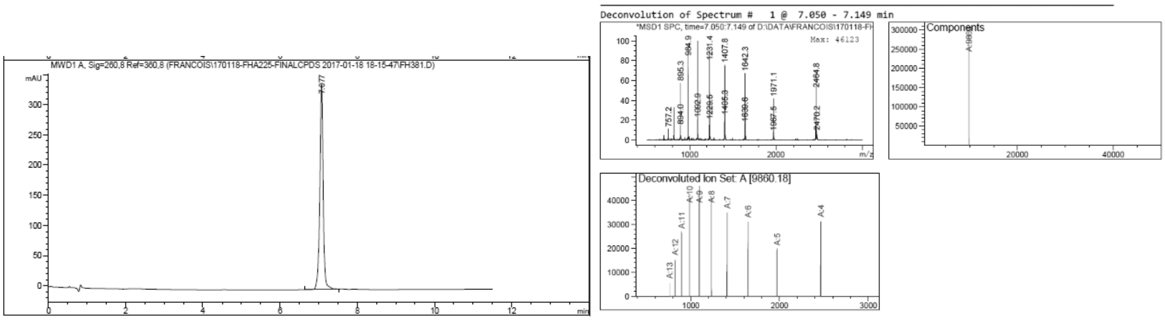

Component	Molecular Weight	Absolute Abundance	Relative Abundance
A	9860.18	286606	100.00

*** End of Report ***

Figure S30. Conjugate **28**

MW (g/mol): calculated 9859.15, found 9860.62; ∆m (%) = 0.02

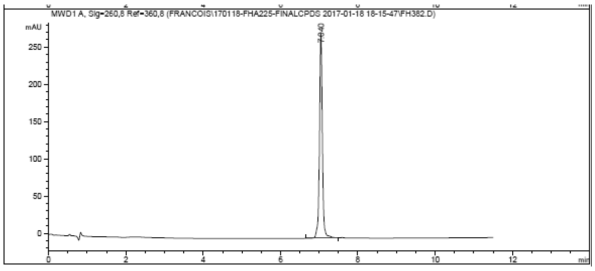

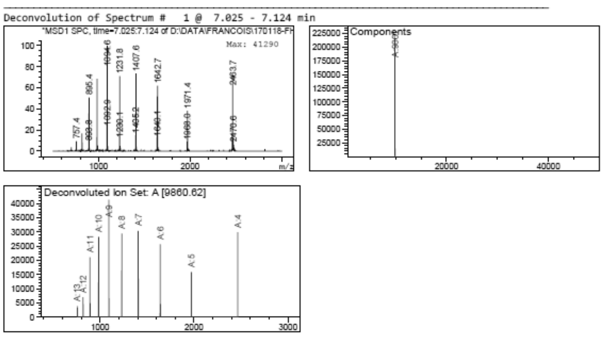

Component	Molecular Weight	Absolute Abundance	Relative Abundance
A	9860.62	213857	100.00

*** End of Report ***
